# Epididymosome‐Supplemented Extender Induces Changes in Morpho‐Functional Traits and microRNA Levels of Post‐Thaw Sperm and Improves Embryo Developmental Potential

**DOI:** 10.1002/mrd.70138

**Published:** 2026-07-30

**Authors:** Laura Gabrielli Haupenthal, Maria Alice de Almeida, Cibele Maria Prado, Amanda Nespolo Silva, Gabriela Melendes Schneider, Paola Maria da Silva Rosa, Flávio Vieira Meirelles, Juliano Coelho da Silveira, Felipe Perecin, Maíra Bianchi Rodrigues Alves

**Affiliations:** ^1^ Department of Veterinary Medicine, School of Animal Science and Food Engineering University of São Paulo Pirassununga São Paulo Brazil; ^2^ Department of Pathology, Theriogenology and One Healthy, School of Agricultural and Veterinary Sciences São Paulo State University Jaboticabal São Paulo Brazil

**Keywords:** epididymis, extracellular vesicles, in vitro fertilization, miRNAs, paternal effect

## Abstract

While molecular signature contributes to sperm quality and fertility potential, sperm may lost key molecules during cryopreservation. Since sperm cells are transcriptionally inert and rely on interactions with epididymosomes (epEVs) to acquire molecules, herein, we investigated the effects of the addition of epEVs to the cryopreservation extender on post‐thaw sperm quality and fertility potential. Epididymal sperm were obtained from six bulls (*n *= 6) and a pool of epEVs was formed from the epididymal fluid of 15 bulls. After, a total of 30 × 10^6^ sperm was cryopreserved in the presence (epEVs) or not (Control) of PKH67 green‐labeled epEVs. Post‐thaw, sperm were evaluated for interaction with epEVs by flow cytometry (FC) and fluorescence microscopy (FM); motility, vigor, and morphology by microscopy; membrane integrity by FC and FM; microRNA levels by qPCR; and fertility potential by in vitro embryo production (IVEP). EpEVs‐sperm showed interaction with green‐epEVs, and higher motility and plasma membrane integrity compared to Control‐sperm. Levels of 28 microRNAs were higher or uniquely detected in epEVs‐sperm. IVEP with epEVs‐sperm resulted in a higher rate of hatched blastocyst. Thus, epEVs‐supplemented to the sperm cryopreservation extender improved post‐thaw sperm quality and embryo developmental potential. These findings provide new insights into the molecular modulation of sperm fertility.

## 1 Introduction

The commercialization of cryopreserved semen in Brazil showed significant growth with an increase of 11.80% in the third quarter of 2025 compared to 2024 (ASBIA 2025). Despite this increase, a loss of approximately 50% in post‐thaw sperm quality is still observed, regardless of the extenders and cryoprotectants used in the protocols (Celeghini et al. 2008; Thomas et al. 1998). Since sperm quality directly impacts fertility, developing sperm cryopreservation protocols that minimize damage to sperm quality is imperative.

Sperm quality is defined by multiple factors, such as the ability of sperm to reach the fertilization site, fertilize the oocyte, and contribute to the development of a high‐quality embryo (reviewed by Alves et al. 2020). In this context, structural and morpho‐functional sperm traits are related to the ability of sperm to reach the fertilization site and fertilize the oocyte (Gillan et al. 2008). Intrinsic features such as DNA integrity as well as the molecular content of sperm, including proteins and noncoding RNAs (ncRNAs), are associated with their ability to contribute to early embryonic development (Alves et al. 2019). Nevertheless, understanding the importance of all these characteristics in determining fertility potential, which defines sperm as healthy, is relatively recent, especially regarding their molecular signature (reviewed by Alves et al. 2020).

Physiologically, sperm are transcriptionally inert, and their molecular signature is regulated through three main processes: during the final stage of spermatogenesis (spermiogenesis) in the testes, throughout sperm maturation in the epididymis, and during the post‐epididymal journey (reviewed by Rangel et al. 2025). Although part of the sperm molecular components are indeed accumulated during spermiogenesis, the main mechanism for their acquisition occurs as sperm pass through different structures of the male and female reproductive tracts, with epididymal maturation being primarily responsible for this molecular regulation (Frenette and Sullivan 2001; Reilly et al. 2016). In the epididymis, sperm arrive from the testes already morphologically formed and undergo the process of sperm maturation, during which they undergo molecular changes and acquire motility and fertilizing ability (Plant and Zeleznik 2015).

It has been reported that sperm acquire protein and ncRNA molecules during their transit through the epididymis *caput*, *corpus*, and *cauda* segments, which are important for sperm quality. The transfer of molecular content occurs primarily through the release of extracellular vesicles (EVs) by the epididymal epithelium, which interact with sperm confined in the epididymal lumen. This communication represents a highly regulated process, as sperm and EVs obtained from different epididymal segments exhibit distinct molecular profiles, including proteins, lipids, and ncRNAs, such as microRNAs (miRNAs) (Belleannée et al. 2013; Girouard et al. 2011; Nixon et al. 2015; Skerget et al. 2015). In particular, sperm‐borne miRNAs are known to be transmitted from sperm to oocytes at the time of fertilization and to influence early embryonic development, including the quality of the first cleavage and the activation of the embryonic genome (Alves et al. 2019; Isacson et al. 2025; Liu et al. 2012; Pinto et al. 2025; Trigg and Conine 2024; Wang et al. 2017; Yuan et al. 2015). Thus, since the intrinsic sperm miRNA signature is capable of influencing sperm fertilizing potential, mainly through its contribution to the zygote at the moment of fertilization, epididymal EVs (epididymosomes) might modulate sperm fertility potential during the cryopreservation process by communicating with sperm.

Currently, research has been conducted to explore the use of EVs derived from seminal plasma, that originated from the testes, epididymis, prostate and other sexual glands, in sperm cryopreservation in bulls (Kowalczyk and Kordan 2024; Shamsi et al. 2024), humans (Gavrilov et al. 2025; Hassani et al. 2025), and dogs (Tanrıkulu et al. 2025), demonstrating positive effects on sperm motility, chromatin integrity, mitochondrial membrane potential, plasma membrane integrity, and post‐thaw molecular levels. However, to the best of our knowledge, no studies have evaluated the role of epididymosomes in this context. Considering that epididymosomes are naturally responsible for transferring specific proteins and RNAs to sperm during their maturation, it is plausible to assume that their supplementation during cryopreservation could provide targeted and physiologically relevant support, thereby contributing to the preservation of fertilizing potential. Since epididymosomes carry miRNAs, such as miR‐148b (Sharma et al. 2016; Reilly et al. 2016) and miR‐34c (Belleannée et al. 2013), which are essential for fertilizing potential and early embryonic development, and considering that the sperm cryopreservation process causes damage to these miRNAs, resulting in their reduced levels in post‐thaw sperm (Ezzati et al. 2021; W.‐M. Liu et al. 2012; Xu et al. 2021), using epididymosomes in the cryopreservation could recover the levels of these miRNAs. However, it remains unclear whether epididymosomes could communicate with sperm during cryopreservation and also modulate sperm molecular content, quality, and fertility potential.

Therefore, the objective of the present study was to investigate the interaction between sperm and epididymosomes obtained from the bovine epididymal *cauda* added to the sperm cryopreservation extender durin*g* the cryopreservation procedures and assess, in post‐thaw samples, the sperm morpho‐functional quality, sperm miRNA profile, as well as the fertility potential, and embryo developmental potential.

## 2 Materials and Methods

### 2.1 Reagents and Chemicals

Unless otherwise stated, all reagents and chemicals used were purchased from Sigma‐Aldrich (St. Louis, MO, USA). The fluorescent probes used were: Hoechst 33342 (Hoechst; reference number H1399), 5,5′,6,6′‐tetrachloro‐1,1′,3,3′‐tetraethylbenzimidazolcarbocyanine iodide (JC‐1; T3168), SYTO 59 (Syto59; S11341), and C11‐BODIPY 581/591 (Bodipy; D3861), purchased from Thermo Fisher Scientific (Waltham, Massachusetts, USA); and propidium iodide (PI; P4170), PKH67 (Green Fluorescent Cell Linker) and *Pisum sativum agglutinin* conjugated to fluorescein isothiocyanate (FITC‐PSA; L0770), purchased from Sigma‐Aldrich.

### 2.2 Obtention of Testicular‐Epididymal Complexes and Experimental Design

A total of 21 pairs of testicular‐epididymal complexes from matured bulls obtained on a local slaughterhouse was used. The pairs from each bull were collected and the epididymal *cauda* was further dissected for the collection of epididymal fluid (EF). Of the collected samples, the EF from 15 bulls was processed for the isolation of epididymosomes (epEVs) that formed a unique pool of epEVs that was stained with a green‐labeled staining previous to cryopreservation. The EF from the remaining six bulls was processed for sperm isolation and the sperm sample from each bull (*n* = 6) was distributed into the treatments: (1) cryopreserved with the pool of epEVs (epEVs group); and (2) cryopreserved in the same conditions in the absence of epEVs (Control group). Immediately before cryopreservation, sperm from the Control and epEVs groups were evaluated for sperm motility and vigor. Following cryopreservation, sperm samples were kept at −196°C until post‐thaw sperm analyses regarding interaction with green‐labeled epEVs, morpho‐functional characteristics (motility, vigor, abnormalities, plasma and acrosome membrane integrity, and mitochondrial membrane potential), molecular characteristics related to microRNA profiles, and fertility potential by *in vitro* embryo production rates. The protocols and guidelines established by the Ethics Committee on Animal Use of the University of São Paulo (number 8312020920) were followed to carry out the procedures.

### 2.3 Collection and Processing of Epididymal Fluid

After collection at the slaughterhouse, the epididymis samples were stored in a box with ice at 4°C until arrival at the laboratory. In the laboratory, the epididymis was isolated from the testicular‐epididymal complex and washed with saline solution (NaCl 0.9%), added with 500 UI/mL of penicillin and 500 μL/mL of streptomycin. After the dissection and isolation of the epididymal *cauda* was performed by isolating the *cauda* by cutting between the *corpus* and the *cauda* of the epididymis, preserving the *vas deferens*. Then, the epididymis *cauda* was washed using the intraluminal perfusion technique with retrograde flow by inserting a 22G catheter into the *vas deferens* to obtain the EF according to Belleannée et al. (2013). Subsequently, with the aid of a 10 mL syringe, PBS (phosphate buffered saline; 8 mg/mL NaCl, 0.2 mg/mL KCl, 1.44 mg/mL Na_2_HPO_4_, and 0.24 mg/mL KH_2_PO_4_) was infused into the lumen of the epididymal *cauda* of each bull, and the EF was collected. Once obtained the EF from both *cauda*, it was mixed and formed a single EF per bull to be processed for isolation pool epEVs or sperm, according to de Almeida et al. (2024).

### 2.4 Isolation and Staining of Epididymosomes Pool

For the isolation of the pool of epEVs, the EF from 15 bulls was collected and processed through a sequential centrifugations/ultracentrifugations protocol from Alves et al. (2021). For this, the first centrifugation was performed at 600 × *g* for 10 min at 4°C to remove sperm. Then, the supernatant underwent two centrifugations at 4000 × *g* for 20 min at 4°C and a final centrifugation at 16,500 × *g* for 30 min at 4°C to remove any remaining cellular debris. At the end of the process, the centrifuged EF from each male was frozen for subsequent isolation of the epEVs pool. For the formation of epEVs pool, the post‐thaw EF from each bull was filtered using a 0.22 μm filter and mix forming a post‐thaw EF pool from the 15 males. A fraction of this pool was first subjected to two ultracentrifugations at 119,700 × *g* for 70 min at 4°C to obtain and characterize the epEVs based on concentration, diameter, morphology, and the presence of specific epididymosomes markers prior to the cryopreservation process. Afterwards, this pool was used to isolate epEVs for the cryopreservation experiment.

The remaining fraction of the EF pool was thawed on the day of cryopreservation to obtain the epEVs pool pellet through two ultracentrifugations (119,700 × *g*/70 min, 4°C). At the end of the isolation, the epEVs pool was stained with PKH67 according to Alves et al. (2026). Subsequently, the stained epEVs were ultracentrifuged at 100,000 × *g* for 30 min at 4°C and resuspended in 90 μL of PBS that was added to the BoviFree extender (Minitube, Germany) to a final concentration of 1000 epididymosomes per sperm, according to Alves et al. (2026). Thereafter, the sperm cryopreservation procedure was conducted. The same staining protocol was applied to PBS, which was stained, ultracentrifuged, resuspended in 90 μL of PBS, and added to the BoviFree extender (Minitube, Germany), used as the control group.

### 2.5 Assessment of Epididymosomes Quality

#### 2.5.1 Nanoparticle Tracking Analysis

For the evaluation of epEVs diameter size and concentration, the epEVs pellet after isolation was diluted 1:200 in PBS and analyzed using the NanoSight NS300 and NanoSight NTA software v3.1 (Malvern Panalytical, Malvern, United Kingdom). For that, epEVs sample was analyzed by recording five 30‐s videos with the camera at level 12, gain 1, and a temperature of 38.5°C. After, the data of the videos were used to assess the mean of the epEVs concentration, as well as the mode of epEVs diameter. Control was performed by analyzing PBS (without epEVs) that was processed and diluted similarly to the epEV sample. Diameter size and concentration of epEVs were calculated after instrument‐based correction for sample dilution.

#### 2.5.2 Transmission Electron Microscopy

For the evaluation of epEVs morphology, the isolated epEVs were diluted in 200 μL of fixative solution (0.1 M Cacodylate; 2.5% Glutaraldehyde; 4% Paraformaldehyde; pH 7.2–7.4) for 2 h at room temperature. After this period, the supernatant of the fixative solution was removed, and 2 mL of PBS was added to the sample. The samples were centrifuged at 119,700 × *g* for 70 min at 4°C. The obtained pellet was resuspended in 50 μL of buffer solution (0.1 M cacodylate) and was placed on a copper grid coated with Pioloform and left for 15 min to allow the excess buffer solution to evaporate. Then, a drop of 2% uranyl acetate was added and left for 3 min. The excess was removed using damp filter paper before analyzing the samples with a transmission electron microscope (FEI 200 kV, model Tecnai 20, LAB6 emitter). Transmission electron microscopy images were also used to determine the diameter of epEVs. For this analysis, epEVs from 12 fields were evaluated, totaling 53 epEVs. Only structures with a morphology similar to epEVs were considered. The diameter size was determined using ImageJ software (NIH; https://imagej.net/ij/) by measuring the width and the length of each structure with the Straight tool, considering the scale bar present in the images. The diameter was estimated as the mean of the width and length measurements.

#### 2.5.3 Flow Cytometry

For assessing the specific markers of epEVs, the flow cytometry was used. Prior to incubation with epEVs, the antibodies were centrifuged at 20,000 × *g* for 30 min at 4°C. epEVs were incubated with FITC‐conjugated monoclonal mouse anti‐CD81 (ab239256, Abcam, UK), anti‐CD9 (ab18241, Abcam, UK), and anti‐CD63 (ab18235, Abcam, UK). For this, 10 µL of the epEV sample diluted in PBS was incubated with 1 µL of the respective antibody for 2 h at room temperature on a shaker. As a negative control, FITC‐conjugated anti‐Calnexin (sc‐23954, Santa Cruz Biotechnology, USA) was used to detect possible cellular contamination. For this, the epEVs were treated with Triton X‐100 (T8787, Sigma‐Aldrich, USA; 1:1) for 15 min at room temperature and incubated with 1 µL anti‐Calnexin for 2 h. After staining, the samples were diluted in 200 µL of PBS for analysis. A total of 50 µL was analyzed using the Cytoflex flow cytometer (Beckman Coulter, USA) with the corresponding filter for the fluorophore (FITC). The cytometer was configured for nanoparticle detection according to the excitation wavelength of the fluorophore conjugated to the antibody, using the violet side scatter (V‐SSC) channel. Gain and threshold values were adjusted according to the manufacturer's recommendations for fluorescent Megamix‐Plus SSC and FSC beads (BioCytex, France), covering sizes from 100 to 900 nm and the fluorescence of interest. The event acquisition rate was set to 2000 events per second, with an abortion rate of up to 5%. Gating was determined based on controls performed using PBS incubated with antibody at the same conditions of epEVs.

### 2.6 Cryopreservation of Epididymal Cauda Sperm With the Epididymosomes

Following collecting of EF from each bull individually (*n* = 6), the samples were evaluated for sperm concentration, by diluting sperm in 4% formaldehyde in PBS (1:100) and counting sperm using a Neubauer chamber under contrast phase microscopy (Nikon, 80i model, Tokyo, Japan) at 400× magnification, and for sperm morphology by fixing the samples in 4% formaldehyde in PBS and classifying 200 sperm cells according to major, minor, and total defects (major plus minor) as described by Barth and Oko (1989), using phase‐contrast microscopy (Axioplan 2 ‐ Carl Zeiss, Oberkochen, Germany) at 1000× magnification in oil. Subsequently, the EF was centrifuged at 600 × *g* for 10 min to isolate the sperm. The supernatant was discarded, and the BoviFree extender (Minitube, Germany) was added, either supplemented (epEVs group) or not (Control group) with the epEVs pool previously stained with PKH67. Once the extender was added, it was kept with the sperm in a water bath for 30 min at 32°C. At the end of this period, subjective motility (%), assessed by analyzing the total movement of sperm in the sample, characterized as the percentage of motile sperm relative to immotile sperm, and vigor: 0 (absent), 1 (slow), 2 (active), 3 (fairly active), 4 (very active), and 5 (vigorous) (CBRA 2013) were evaluated using contrast phase microscopy at 100× magnification.

Subsequently, sperm doses were loaded into 0.25 mL straws containing 30 × 10^6^ sperm per dose, sealed with polyvinyl alcohol, and placed on the rack of the TK4000 machine (TK Tecnologia, Uberaba, MG, Brazil). For performing the cooling rate of the refrigeration curve, equilibrium time and cryopreservation, the TK4000 machine was programmed with the following protocol: a cooling rate of 0.5°C/min, an equilibrium time of 2 h 30 min at 5°C, and a freezing curve of 20°C/min, followed by immersion of the straws in liquid nitrogen (−196°C) and transfer to the cryogenic tank for storage at −196°C according to the protocol described in de Almeida et al. (2024). The sperm samples used showed a mean ± standard error of the mean (SEM) of sperm concentration of 488.33 ± 24.09 × 10^6^ sperm/mL, with 50.08 ± 1.98% major defects, 43.17 ± 1.62% minor defects, and 93.25 ± 0.45% total defects.

### 2.7 Evaluation of the Interaction Between Epididymosomes and Sperm

Flow cytometry and fluorescence microscopy were applied for the evaluation of the interaction between sperm and epEVs. Regarding flow cytometry, a BD Accuri C6 device (BD Biosciences, San Jose, CA, USA) was used, configured with two argon lasers for sample excitation (blue: 488 nm; red: 635 nm). SSC (Side Scatter: cell granularity) and FSC (Forward Scatter: cell size) characteristics were used, as well as two filters (FL1: 533/30 nm for PKH67 fluorescence detection; FL4: 675/25 nm for SYTO59 fluorescence detection) for the analyses. For the analyses, post‐thaw sperm from Control and epEVs groups from each bull were diluted to a final concentration of 5 × 10^6^ sperm/mL in 150 μL of TALP sperm medium and incubated with 1 μL of SYTO‐59 probe 750 nM. The samples were then incubated for 8 min at 37°C and subsequently diluted to a final concentration of 2.5 × 10^6^ sperm/mL in TALP sperm medium (4.2 mg/mL NaCl, 1.87 mg/mL KCl, 2.1 mg/mL NaHCO_3_, 0.05 mg/mL NaH_2_PO_4_, 0.145 mg/mL CaCl_2_H_2_O, 0.08 mg/mL MgCl_2_·6H_2_O, 6.5 mg/mL Hepes) supplemented with 5 mg/mL glucose, 18.50 mL sodium lactate, 140 mg/mL sodium pyruvate and 200 mg/mL bovine serum albumin, for flow cytometry analysis. A total of 20,000 SYTO59‐positive events, representing the sperm population, were recorded. Within this population, sperm exhibiting higher or lower PKH67 fluorescence intensity were analyzed, and the median green fluorescence intensity per cell was used to assess the interaction between sperm and PKH67‐stained particles.

For the evaluation of sperm‐epEVs interaction by fluorescence microscopy, post‐thaw sperm from Control and epEVs groups were thawed and diluted in 150 μL of TALP sperm medium, to a final concentration of 10 × 10^6^ sperm/mL and stained with 2 μL of Hoechst 33342 10 μg/mL. The sample was incubated for 8 min at 37°C, and sperm were classified as positive or negative for the presence of green fluorescent spots (PKH67) using epifluorescence microscopy (Axioplan 2—Carl Zeiss, Oberkochen, Germany) at 1000× magnification with oil immersion, counting 200 sperm cells per sample. Images were also captured using the epifluorescence microscopy (Thunder Imager 3D Assay; Leica), at 630× magnification to visualize the green fluorescence of PKH67 on sperm cells.

### 2.8 Assessment of Post‐Thaw Sperm Features

For the evaluation of advanced sperm morpho‐functional characteristics, two straws of cryopreserved sperm from the Control and epEVs groups of each bull were thawed in a water bath at 37°C for 30 s and transferred to preheated microtubes. The samples were then diluted in TALP sperm medium and evaluated for motility by subjective method and using a computer‐assisted sperm analysis system (CASA), for plasma and acrosomal membrane integrity and mitochondrial membrane potential by fluorescence microscopy, and for plasma membrane integrity and mitochondrial membrane potential by flow cytometry.

#### 2.8.1 Sperm Motility

Sperm motility characteristics were evaluated subjectively as previously described and using a CASA system with the SCA software (Sperm Class Analyzer; Microptic, Barcelona, Spain). For this, 8 μL of sperm samples diluted to a concentration of 20 × 10^6^ sperm/mL in TALP sperm medium were placed in a Makler chamber (Selfi‐Medical Instruments, Haifa, Israel) for analysis. The chamber was coupled to a microscope at 100x magnification, and at least 500 sperm cells were analyzed for the following motility parameters: total motility (%), progressive motility (%), rapid sperm (%), progressive velocity (VSL, μm/s), curvilinear velocity (VCL, μm/s), path velocity (VAP, μm/s), linearity (LIN, %), straightness (STR, %), wobble (WOB, %), lateral head displacement (ALH, μm/s), beat‐cross frequency (BCF, Hz), and percentage of hyperactivated sperm parameters as standardized by Ryu et al. (2019) and Turri et al. (2012).

#### 2.8.2 Sperm Plasma and Acrosome Membrane Integrity and Mitochondrial Membrane Potential by Fluorescence Microscopy

For the analysis of sperm plasma, acrosome, and mitochondrial membranes, sperm were diluted to a final concentration of 10 × 10^6^ sperm/mL in 150 μL of TALP sperm medium and stained with 2 μL of Hoechst 33342 10 μg/mL, 2 μL of propidium iodide 0.5 mg/mL, 6 μL of JC‐1 153 μM, and 20 μL of FITC‐PSA 100 μg/mL. The samples were incubated for 8 min at 37°C and analyzed using an epifluorescence microscope (Axioplan 2—Carl Zeiss, Oberkochen, Germany) at 1000× magnification with oil immersion. For evaluation, 200 sperm cells per sample were classified as follows: integrity of plasma membrane (%), integrity of acrosome membrane integrity (%), high mitochondrial membrane potential (%), integrity of plasma and acrosome membranes (%), and sperm cells with integrity of plasma and acrosome membranes and high mitochondrial membrane potential (PIAIA sperm, %). In addition, sperm were analyzed for sperm plasma membrane integrity by epifluorescence microscopy (Thunder Imager 3D Assay; Leica) at 630× magnification to visualize the green fluorescence of PKH67 on sperm cells with plasma membrane integrity (Supplementary Figure S1).

#### 2.8.3 Sperm Plasma Membrane Integrity and Mitochondrial Membrane Potential By Flow Cytometer

For the evaluation of plasma membrane integrity and mitochondrial membrane potential, a BD Accuri C6 flow cytometer (Becton‐Dickinson, San Jose, CA, USA) was used, equipped with an argon laser (488 nm) and a red laser (635 nm), both employed to excite the samples. Four filters were used in the analyses: 533/30 nm, 585/40 nm, 675/25 nm, and > 670 nm (Alves et al. 2019). Sperm concentration was adjusted to 5 × 10^6^ sperm/mL in TALP sperm medium. Samples were incubated with 1 μL of SYTO59, 1 μL of propidium iodide 0.5 mg/mL, and 1 μL of JC‐1 153 μM for 10 min at 37°C. A total of 20,000 events identified as sperm were analyzed and classified according to the percentage of sperm with integrity of plasma membrane, sperm with integrity of plasma membrane and with high mitochondrial membrane potential, as well as the median fluorescence intensity of the mitochondrial membrane potential.

### 2.9 Levels of microRNAs and Functional Analysis

The relative levels of 380 bovine miRNAs were evaluated by reverse transcription quantitative polymerase chain reaction (RT‐qPCR) in sperm as described by Alves et al. (2019). For this, one sperm straw from the Control group and one from the epEVs group of three bulls presenting a similar sperm quality regarding motility were thawed and subjected to two centrifugations at 600 × *g* for 5 min. After, the sperm pellet was resuspended in nuclease‐free water for total RNA extraction and purification. RNA extraction was performed using RNAzol according to the manufacturer's instructions. RNA concentration and purity were determined using a NanoDrop 1000 spectrophotometer (Thermo Fisher Scientific).

The RT‐qPCR was performed with 200 ng of the resulting cDNA, using SYBR Green (Qiagen, Hilden, Germany), specific forward primers (Supplementary File S1), and a universal reverse primer (Qiagen). The reaction was run on a QuantStudio 6 Flex (Thermo Fisher Scientific) under the following cycling conditions: 95°C for 15 min, followed by 45 cycles of 94°C for 15 s, 55°C for 30 s, and 70°C for 30 s. The melting curve was used to confirm the amplification of the product. Reactions with Ct values above 37 or with three or more peaks in the dissociation curve were excluded. The miRNAs bta‐miR‐211, bta‐miR‐764, and bta‐miR‐96b, consistently detected across groups according to NormFinder, were used to calculate relative levels. The ΔCt method was employed to determine the relative expression of each individual miRNA (Schmittgen and Livak 2008). Subsequently, the values were transformed using the 2^‐∆ct^ calculation and used for figure generation. Exclusive miRNA detection was based on the presence of the miRNA in all three bulls within one group, associated with its non‐detection in all three bulls of the other group.

The exclusive miRNAs from sperm of epEVs group and the differentially detected miRNAs between epEVs and Control groups were analyzed using the MiRWalk software, and the biological functions of the genes associated with miRNAs were identified through the DAVID tool. For the present study, the first 20 most significantly enriched biological functions were considered, ranked according to the −log_10_ of the *p*‐value, based on functional annotation.

### 2.10 In Vitro Embryo Production

For *in vitro* embryo production, the conditions for *in vitro* maturation and fertilization were 38.5°C with 5% CO_2_ in air and high humidity, while the conditions for *in vitro* culture were 38.5°C with 5% CO_2_, 5% O_2_, 90% N_2,_ and high humidity. Initially, follicular fluid was aspirated from ovaries obtained from a slaughterhouse, which contained follicles with diameters ranging from 3 to 6 mm. After aspiration, the follicular fluid was processed for the retrieval of cumulus–oocyte complexes (COCs) under a stereomicroscope (SMZ 745T model, Nikon), and only COCs classified as grade I or II were selected. *In vitro* maturation was performed in TCM 199 maturation medium supplemented with 26 mM sodium bicarbonate, 10% fetal bovine serum (FBS), 0.2 mM sodium pyruvate, 50 µg/mL gentamicin, 0.5 µg/mL follicle‐stimulating hormone (FSH; Folltropin), and 50 µg/mL human chorionic gonadotropin (hCG; Vetecor). The COCs were cultured in 35 mm plates containing maturation medium for 22 h.

For the *in vitro* fertilization (IVF) stage, three cryopreserved sperm straws from the Control and epEVs groups of each bull were used. The semen samples were processed through a Percoll gradient (45% and 90%) and centrifuged at 3600 × *g* for 7 min. After separation, the material was washed and centrifuged again at 520 × *g* for 5 min. The resulting pellet was resuspended in IVF medium—Tyrode's lactate base supplemented with 6 mg/mL BSA, 22 μg/mL sodium pyruvate, 5.5 IU/mL heparin, 40 μL/mL PHE (2 mM d‐penicillamine, 1 mM hypotaurine, and 245 μM epinephrine), and 50 μg/mL gentamicin. The final suspension was adjusted to a concentration of 1 × 10^6^ sperm/mL and used for insemination of 100 μL droplets containing 20–25 oocytes in IVF medium, followed by 18 h of incubation. After this period, the presumptive zygotes were transferred to *in vitro* culture plates containing SOF medium supplemented with amino acids, citrate, and inositol, as well as 5 mg/mL BSA, 2.5% FBS, 22 μg/mL sodium pyruvate, and 50 μg/mL gentamicin. The culture was maintained for 7 days from fertilization, and first cleavage rates were assessed at 28 hpi (hours post‐insemination), cleavage at 96 hpi, and blastocyst formation at 168 hpi. Blastocysts were classified as early blastocyst (EB), typical blastocyst (BL), expanded blastocyst (BX), or hatched blastocyst (HB), according to the International Embryo Transfer Society (Stringfellow and Givens 2010).

### 2.11 Evaluation of Blastocyst Cell Number

Blastocyst embryos were collected and washed, fixed in 4% PFA in PBS containing 0.1% polyvinylpyrrolidone (PVP) for 15 min, and permeabilized for 30 min in 1% Triton X‐100 solution. Afterward, the structures were washed in PBS with 0.1% PVP and stained with 10 μg/mL Hoechst 33342 for 15 min. The embryos were then washed again, and the slides were mounted with Prolong Antifade (Thermo Fisher Scientific). Images were acquired using a fluorescence microscope (Thunder Imager 3D Assay; Leica) the following settings: brightfield 550 nm, 50 ms and Hoechst LED405, 450 nm, 10 ms, under 400× magnification and analyzed using ImageJ software (NIH; https://imagej.net/ij/), as described by (Alves et al. 2019).

### 2.12 Statistical Analysis

Interaction of epEVs‐sperm, sperm morpho‐functional traits, number of cells in blastocyst, and sperm microRNA levels were evaluated using the MIXED procedure of the SAS 9.3 program (Statistical Analysis System) through Analysis of Variance. For that, first, the data were subjected to a normality test using the Shapiro–Wilk test. When necessary, data were either transformed or outliers were excluded. For *in vitro* embryo production rates, evaluation was performed using the chi‐square frequency test. The significance was considered when *p*≤ 0.05.

## 3 Results

We investigated the effects of the supplementation of the cryopreservation extender with epididymosomes (epEVs) on sperm quality. For that, once the pool of epEVs was formed and characterized, sperm were cryopreserved with green‐labeled epEVs. Following confirmation of sperm interaction with epEVs, post‐thaw sperm were analyzed regarding morpho‐functional traits, miRNA signature, and fertility potential.

### 3.1 Epididymosomes Were Efficiently Isolated From Epididymal Fluid

**Figure 1:**
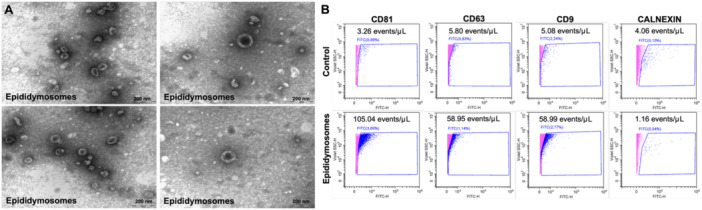
Assessment of epididymosomes quality. In (A), transmission electron microscopy of extracellular vesicles showing the morphology of epididymosomes. Scale bar: 200 nm. In (B), gates showing the positive event count per µL for fluorescence of each marker (CD81, CD63, CD9, Calnexin) in control (i.e., absence of epididymosomes) and epididymosomes pool sample.

Following formation of the pool of epEVs, epEVs were characterized regarding diameter size, concentration, morphology, and specific protein markers. Regarding diameter and concentration analyzed by nanoparticle tracking analysis, the epEVs showed a mode diameter of 114.60 ± 5.10 nm and 60.03 × 10^9^ ± 7.80 × 10^9^ particles/mL of concentration in the pool of epEVs. The morphology of the epEVs confirmed EVs presence by detecting structures with “cup‐shaped” morphology (Figure 1A). Based on the transmission electron microscopy images, epEVs showed a diameter of 100.64 ± 3.04 nm (mean) and 85 nm ± 3.50 (mode). Finally, the epEVs displayed higher levels of specific epididymosomes protein markers (CD81, CD63, and CD9) compared to samples containing no epididymosomes (Control). In addition, detection of calnexin in epEVs, a chaperone protein located in the endoplasmic reticulum that is present in cells, revealed similar levels to samples containing no epididymosomes, indicating no cellular contamination during collection (Figure 1B). Thus, the epEVs were efficiently obtained from the epididymal fluid to form the pool of epEVs from 15 bovine epididymal *cauda*.

### 3.2 Sperm Interacted With Epididymosomes During Cryopreservation

**Figure 2:**
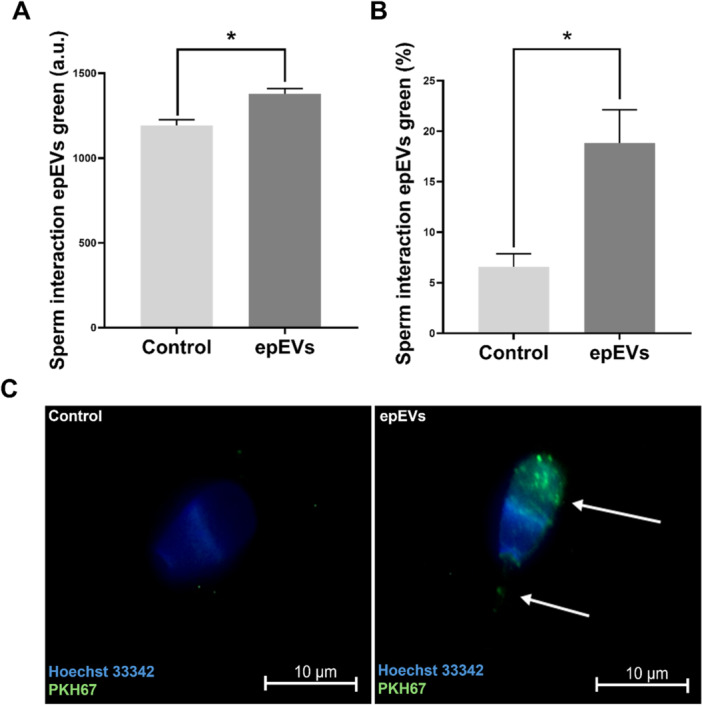
Mean and SEM of the interaction between cryopreserved epididymal *cauda* sperm and epididymosomes of post‐thaw sperm cryopreserved in the absence (Control group) or presence of epididymosomes (epEVs group). In (A), sperm interaction is shown based on the intensity of green fluorescence detected in sperm. In (B), the percentage of sperm positive to green fluorescence spots by fluorescence microscopy is shown. Asterisks indicate significant differences (*p*≤ 0.05). a.u.: arbitrary units. In (C), photomicrograph obtained by epifluorescence microscopy (Axioplan 2–Carl Zeiss, Oberkochen, Germany) at 1000x magnification of post‐thaw sperm cryopreserved in the absence (Control group) or presence of epididymosomes (epEVs group). The arrows indicate the presence of epididymosomes in the sperm. Scale bar: 10 μm.

**Figure 3:**
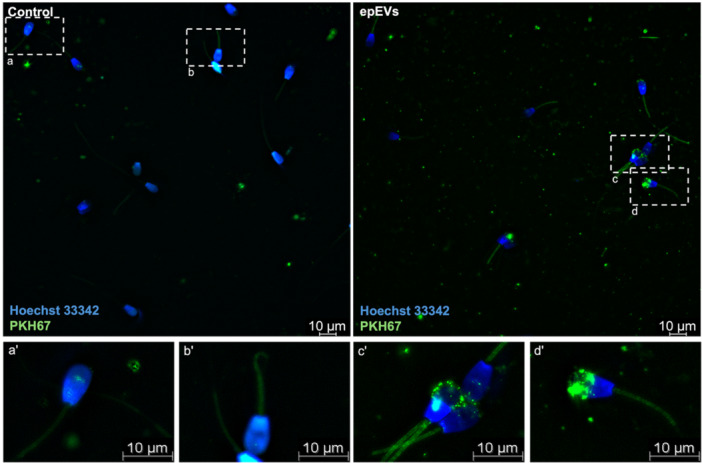
Photomicrograph obtained by epifluorescence microscopy (Thunder Imager 3D Assay; Leica) at 630× magnification of post‐thaw sperm cryopreserved in the absence (Control group) or presence of epididymosomes (epEVs group). In ***a*** and ***b***, sperm without the presence of epididymosomes. In ***c*** and ***d***, sperm that interacted with epididymosomes. Scale bar: 10 μm.

Following characterization, epEVs were stained and added to the cryopreservation extender. Sperm cells were cryopreserved with green‐labeled epEVs and after thawing, sperm were analyzed on flow cytometry for detection of green fluorescence. In that regard, post‐thaw sperm from the epEVs group showed a higher green fluorescence intensity (1325.67 ± 58.29 a.u.) compared to the Control group (1152.17 ± 48.69 a.u.) (*p *= 0.0008; Figure 2A). The percentage of post‐thaw sperm that interacted with epEVs was also assessed by fluorescence microscopy that showed a higher percentage (*p *= 0.01) of green fluorescent spots (PKH67) in the epEVs group (18.85 ± 3.27%) compared to the Control group (6.58 ± 1.29%; Figure 2B,C). The presence of epEVs was noted more frequently in the region of the spermatic head and secondarily in the region of the middle piece, as illustrated in Figures 2C and 3. Correlation analysis showed 76.92% correlation between the evaluation of fluorescence microscopy and flow cytometry.

### 3.3 Sperm Cryopreserved With Epididymosomes Displayed a Higher Post‐Thaw Sperm Motility and Plasma Membrane Integrity

After the incubation period with the extender and immediately previous to cryopreservation, subjective motility and vigor were assessed and displayed similar (*p*> 0.05) parameters between the Control and epEVs groups (Table 1). Subsequently, post‐thaw sperm from both groups were compared regarding morpho‐functional characteristics. Regarding post‐thaw sperm vigor and major, minor, and total defects, no statistical differences (*p*> 0.05) were observed between the Control and epEVs groups (Table 2). However, post‐thaw sperm of epEVs group displayed a higher (*p *= 0.02) subjective motility compared to post‐thaw sperm of Control group according to Table 2.

**Table 1:** Mean, standard error of the mean (SEM) and *p*‐value of subjective motility and vigor of fresh sperm incubated with the extender in the absence (Control group) or presence (epEVs group) of epididymosomes before cryopreservation.

Morpho‐functional traits of fresh sperm (before cryopreservation)	Groups		*p*‐value
	Control (*n*=6)	epEVs (*n*=6)	
Subjective motility (%)	50.83 ± 4.16	53.33 ± 4.94	0.36
Vigor (1–5)	2.33 ± 0.10	2.66 ± 0.16	0.10

**Table 2:** Mean, standard error of the mean (SEM) and *p*‐value of motility and abnormalities traits, integrity of plasma and acrosome membranes and mitochondrial membrane potential assessed by fluorescence microscopy, and integrity of plasma membrane and mitochondrial membrane potential assessed by flow cytometry, of post‐thaw sperm cryopreserved in the absence (Control group) or presence (epEVs group) of epididymosomes. Different letters in the same line indicate a statistical difference (*p*≤ 0.05). a.u.: arbitrary units.

Morpho‐functional traits of post‐thaw sperm (after cryopreservation)	Groups		*p*‐value
	Control (*n*=6)	epEVs (*n*=6)	
Motility and abnormalities traits			
Subjective motility (%)	33.33 ± 4.01^b^	47.50 ± 5.12^a^	0.02
Vigor (1–5)	2.33 ± 0.16	2.50 ± 0.18	0.17
Major defects (%)	31.91 ± 3.66	37.41 ± 5.44	0.14
Minor defects (%)	35.50 ± 9.33	30.44 ± 6.90	0.14
Total defects (%)	67.41 ± 8.78	67.93 ± 9.19	0.84
Integrity of plasma and acrosome membranes and mitochondrial membrane potential assessed by fluorescence microscopy			
PIAIA^a^ (%)	12.81 ± 2.48	17.31 ± 2.72	0.18
Plasma membrane integrity (%)	32.37 ± 6.91	48.07 ± 9.54	0.17
Acrosome membrane integrity (%)	34.34 ± 5.64	35.71 ± 5.93	0.62
High mitochondrial membrane potential (%)	91.42 ± 1.60	87.21 ± 3.34	0.15
Plasma and acrosome membranes integrity (%)	13.48 ± 2.50	18.47 ± 2.88	0.13
Integrity of plasma membrane and mitochondrial membrane potential assessed by flow cytometry			
Plasma membrane integrity (%)	28.50 ± 6.77^b^	39.92 ± 6.52^a^	0.05
Plasma membrane integrity and high mitochondrial membrane potential (%)	23.54 ± 5.99	32.09 ± 6.13	0.08
High mitochondrial membrane potential (a.u.)	21733.67 ± 2759.43	23404.33 ± 2557.92	0.39

^a^ PIAIA: sperm cells with integrity of plasma and acrosomal membranes and high mitochondrial membrane potential.

**Figure 4:**
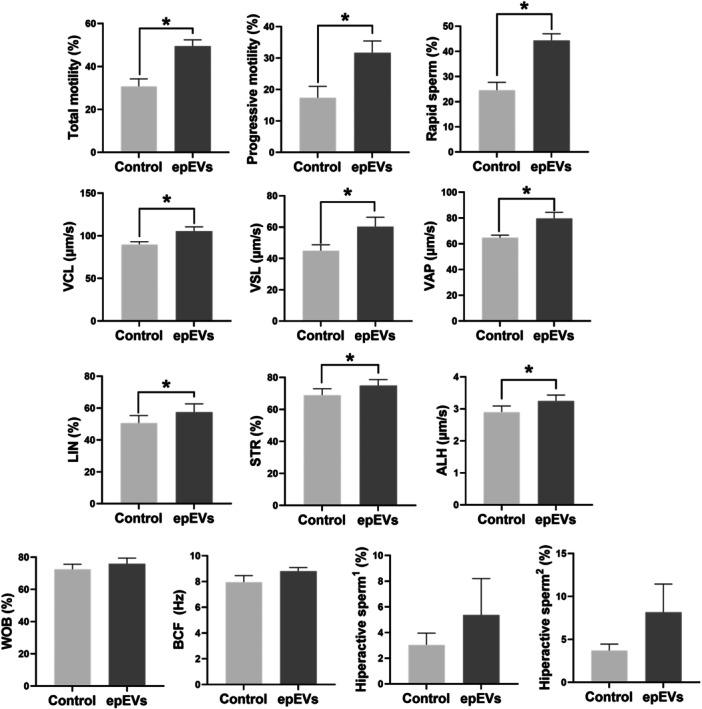
Mean and SEM of the post‐thaw sperm motility parameters evaluated by Computer‐Assisted Sperm Analysis (CASA) of sperm cryopreserved in the absence (Control group) or presence of epididymosomes (epEVs group). Asterisks indicate significant differences (*p*≤ 0.05). ^1^Hyperactive sperm according to Turri et al. (2012): VCL> 70 µm/s, ALH> 5 µm, and VSL˂30%. ^2^Hyperactive sperm according to Ryu et al. (2019): VCL≥ 150 µm/s, ALH≥ 5 µm/s, and VSL≤ 50%.

Regarding motility kinetic parameters, post‐thaw sperm from the epEVs group showed a higher (*p *< 0.05) percentage of sperm with total motility compared to the Control group, as well as higher values for progressive motility, rapid sperm, VCL, VSL, VAP, LIN, STR, and ALH, as shown in Figure 4. The analysis of hyperactivated sperm movement was also performed, based on parameters described by Ryu et al. (2019) and Turri et al. (2012), with these characteristics being similar between the groups (Figure 4). The detailed mean, SEM, and *p*‐value for each motility kinetic parameter are presented in Supplementary Table S1.

Finally, post‐thaw sperm from the epEVs group displayed a similar percentage of plasma membrane integrity, acrosomal membrane integrity, and mitochondrial membrane potential evaluated by fluorescence microscopy compared to sperm from the control group. However, the percentage of sperm with plasma membrane integrity evaluated by flow cytometry was higher (*p *= 0.05) in post‐thaw sperm from the epEVs group compared to the Control group, as shown in Table 2. Regarding the number of sperm with plasma membrane integrity that interact with green‐labeled epEVs, the analysis of sperm batch from one bull showed that 27% of sperm with plasma membrane integrity interacted with epEVs, while 14% of sperm with plasma membrane damage interacted with epEVs (Supplementary Figure S1).

### 3.4 Epididymosomes Added to the Extender Changed Sperm microRNA Epigenome

**Figure 5:**
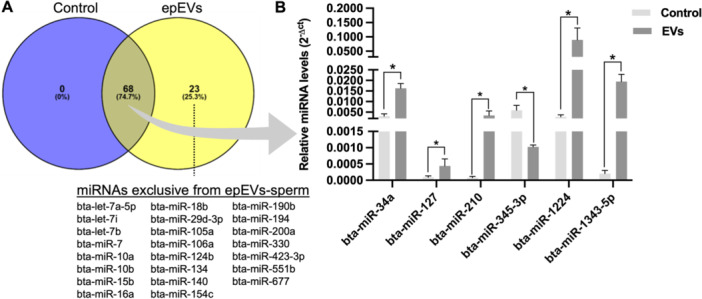
MicroRNAs (miRNAs) detected in post‐thaw sperm cryopreserved in the absence (Control group) or presence of epididymosomes (epEVs group). In (A), Venn diagram showing the presence of 91 miRNAs in post‐thaw sperm of Control and epEVs group, with 23 miRNAs exclusive to sperm of epEVs group and 68 miRNAs detected in sperm from Control and epEVs groups. In (B), mean and standard error of the mean (SEM) of 6 out of 68 miRNAs that showed statistical difference between sperm from Control and epEVs groups with five up‐regulated and 1 down‐regulated in the epEVs group compared to the Control group. Asterisk indicates significant differences (*p *≤ 0.05).

**Figure 6:**
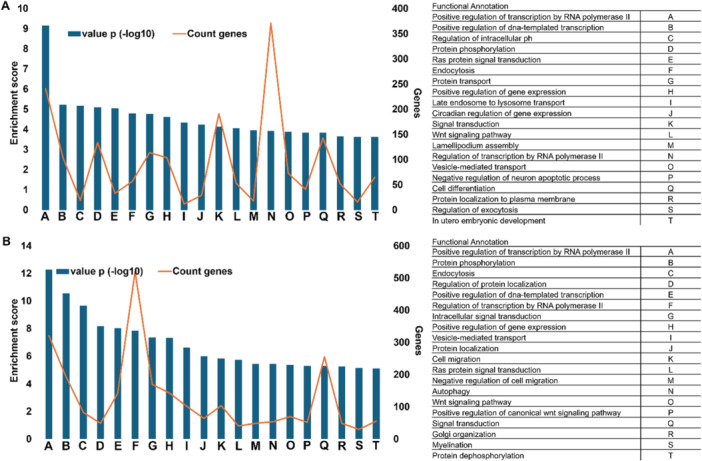
Analysis of the biological functions of the target‐genes of the microRNAs (miRNAs) differently detected in post‐thaw sperm cryopreserved in the absence (Control group) or presence of epididymosomes (epEVs group). In (A), biological functions of the target‐genes of the five up‐regulated miRNAs in sperm from epEVs group. In (B), biological functions of the target‐genes of the 23 exclusive miRNAs in sperm from epEVs group.

Following assessment of sperm morpho‐functional aspects, sperm samples with similar motility in Control and epEVs groups were selected (Supplementary Table S2) to verify the post‐thaw sperm signature of miRNAs. Out of 380 miRNAs investigated in the study, 91 were detected in post‐thaw sperm from Control and/or epEVs group. Out of 91 detected, 23 (bta‐miR‐7a‐5p, −7i, −7b, −7, −10a, −10b, −15b, −16a, −18b, −29d‐3p, −105a, −106a, −124b, −134, −140, −154c, −190b, −194, −200a, −330, −423‐3p, −551b, and −677) were exclusively detected on sperm from epEVs group. Interestingly, none of the 91 detected miRNAs were exclusively detected in sperm from Control group. The major part of the 91 miRNAs, that is, 68 miRNAs were common detected in sperm from Control and epEVs groups (Figure 5A). Among the 68 miRNAs detected in both groups, five (bta‐miR‐34a, −127, −210, −1224, and −1343‐5p) were detected up‐regulated, and 1 (bta‐miR‐345‐3p) was detected down‐regulated in sperm from epEVs group (Figure 5B). The top 20 enriched biological functions related to the up‐regulated miRNAs and exclusive miRNAs are shown in Figure 6A,B. Regarding the functional annotation, the up‐regulated miRNA target genes were related with regulation of transcription, regulation of intracellular pH, protein phosphorylation, and others, while the exclusive miRNA target genes were also involved with regulation of transcription, and protein phosphorylation. A complete list of the biological functions of the target genes of up‐ and down‐regulated miRNAs was included in Supplementary File S2.

### 3.5 Sperm Cryopreserved With Epididymosomes Triggered Higher Rates of Hatching Blastocyst

**Figure 7:**
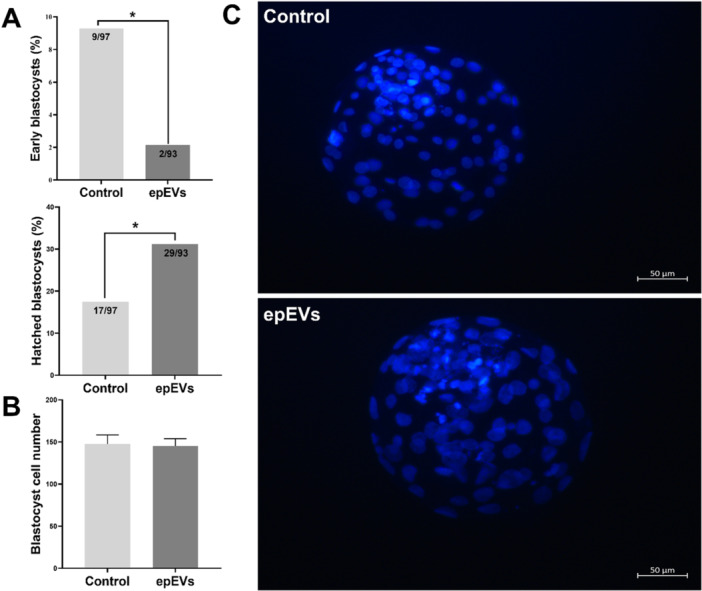
Developmental stages of blastocysts produced with post‐thaw sperm cryopreserved in the absence (Control group) or presence of epididymosomes (epEVs group). In (A), developmental rates of early blastocysts and hatched blastocysts produced with post‐thaw sperm cryopreserved of Control and epEVs groups. In (B), mean and SEM of the number of cells in blastocysts collected from six rounds of embryo production with post‐thaw sperm of the Control and epEVs groups totalizing 21 embryos of Control and 21 of epEVs group. In (C), photomicrograph obtained by epifluorescence microscopy of blastocysts stained with Hoechst 33342 produced with post‐thaw sperm cryopreserved of Control and epEVs groups. Scale bar: 50 μm. Asterisks indicates significant differences (*p *≤ 0.05).

Regarding sperm fertility potential, similar rates (*p *> 0.05) of first cleavage, cleavage, and blastocyst were observed in the Control and epEVs groups (Table 3). However, when examining specifically blastocyst classifications, embryos from Control group (9.28%, 9/97) displayed a higher rate (*p* = 0.03) of early blastocysts compared to the epEVs group (2.15%, 2/93). On the other hand, a higher rate (*p* = 0.02) of hatched blastocysts was found in embryos from epEVs group (31.18%, 29/93) compared to embryos from Control group (17.52%, 17/97) (Figure 7A and Supplementary Figure S2). Similar rates were observed for typical blastocysts (*p* = 0.85; control: 8.25%, 8/97; EVs: 7.53%, 7/93) and expanded blastocysts (*p* = 0.40; control: 64.95%, 63/97; EVs: 59.14%, 55/93). The number of cells counted in the blastocysts showed no statistical differences (*p *= 0.89) between the groups (Figure 7B,C).

**Table 3:** Pre‐implantational developmental rates of embryos produced with post‐thaw sperm characteristics using post‐thaw sperm cryopreserved in the absence (Control group) or presence (epEVs group) of epididymosomes. Statistical difference was considered at *p*≤ 0.05. N: Numbers of positive structures to first cleavage, cleavage, and blastocyst. Rate was calculated by N divided by the total number of oocytes.

Groups	Oocyte	First cleavage*		Cleavage**		Blastocyst***	
		*N*	Rate (%)	*N*	Rate (%)	*N*	Rate (%)
Control (*n *= 6)	318	141	44.34	187	58.81	97	30.50
epEVs (*n *= 6)	321	140	43.61	196	61.06	93	28.97

* *p*‐value = 0.85;

** *p*‐value = 0.56;

*** *p*‐value = 0.67.

## 4 Discussion

**Figure 8:**
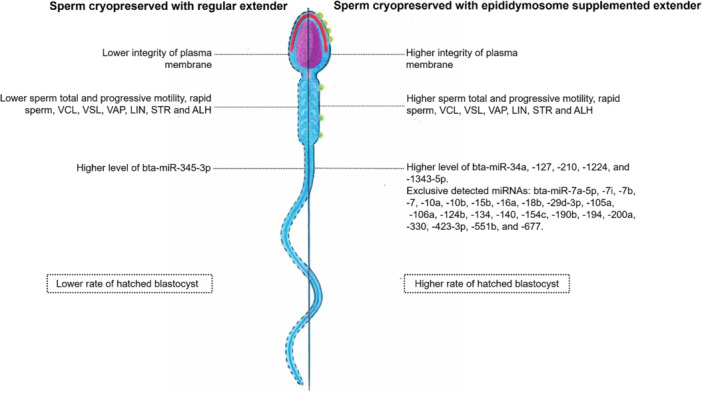
Schematic figure demonstrating the effects of supplementation with epididymosomes to cryopreservation extender on post‐thaw epididymal *cauda* sperm. The figures show that in the presence of epididymosomes during cryopreservation, higher sperm motility parameters (total and progressive motility, rapid sperm, VCL, VSL, VAP, LIN, STR, and ALH), integrity of the plasma membrane, and rate of hatched blastocyst were observed. In addition, sperm cryopreserved with epididymosomes showed lower expression of bta‐miR‐345‐3p, and higher expression of five microRNAs (bta‐miR‐34a, −127, −210, −1224, and −1343‐5p), as well as 23 exclusive miRNAs (bta‐miR‐7a‐5p, −7i, −7b, −7, −10a, −10b, −15b, −16a, −18b, −29d‐3p, −105a, −106a, −124b, −134, −140, −154c, −190b, −194, −200a, −330, −423‐3p, −551b, and −677).

The objective of this study was to investigate the interaction between sperm and epididymosomes during sperm cryopreservation and its effects on post‐thaw sperm quality and fertility potential. In general, our results confirmed that sperm interact with epididymosomes during cryopreservation, which trigger favorable effects to post‐thaw sperm motility and plasma membrane integrity, reprogram the sperm miRNA signature, and increase the production of hatched blastocysts (Figure 8).

First, the obtained epididymosomes were evaluated to verify if they showed similar characteristics of nanoparticles isolated from the *cauda* epididymis. Regarding the diameter, the size of 114.60 ± 5.10 nm of the epEVs measured by nanoparticle tracking analysis in our study displayed a value consistent with the typical size of epididymosomes (50–250 nm) described in literature (Frenette et al. 2010). Interesting, considering the concentration of epEVs (60.03 × 10^9^ particles/mL) and sperm concentration (488 × 10^6^ sperm/mL) of the epididymal fluid used in this study, the proportion of epEVs/sperm found here (123.01 epEVs/sperm) is similar to a previous study of our group (Alves et al. 2026). In addition, epEVs showed a cup‐shaped morphology that was also related in other studies with bovine epididymosomes (Belleannée et al. 2013; Frenette et al. 2010; Girouard et al. 2011). Finally, it was detected the tetraspanins CD9, CD63, and CD81 in epEVs; these proteins are classical markers present on the membranes of EVs (Kowal et al. 2016), including epididymosomes. Usually, the protein markers of epEVs are investigated using Western Blot technique. However, flow cytometry is also an important tool that can be efficiently employed for this characterization since using the proper controls (Théry et al. 2018). Girouard et al. (2011) and Caballero et al. (2013) highlighted the presence of the tetraspanin CD9 in *cauda* epididymosomes in bovines. Thimon et al. (2008) and Luo et al. (2024) demonstrated the existence of CD63 in human epididymosomes. Meanwhile, Batra et al. (2025) and Y. Liu et al. (2019) reported the presence of CD81 in *cauda* epididymosomes in mice. Thus, the epEVs features were consistent with those of epididymosomes. However, considering that the isolation of the epEVs was performed by ultracentrifugation and that this method does not avoid the whole removal of free proteins and other non‐vesicular contaminants (Welsh et al. 2024), the isolated epEV samples should be characterized as an epididymosome‐enriched fraction.

Once characterized, epididymosomes were stained with a green‐fluorescent marker and were supplemented to cryopreservation extender that was used to cryopreserve *cauda* epididymal sperm of six different bulls. First, interaction of sperm with green‐epididymosomes was confirmed after cryopreservation by detecting green fluorescence in post‐thaw sperm by flow cytometry and fluorescence microscopy. However, green fluorescence was also observed in sperm from the control group, which may be related to dye aggregation, since the staining used (PKH67), although previously inactivated (da Silveira et al. 2017), was added to the control group in its free form. Nevertheless, despite the presence of residual free staining in the control group, it is possible to distinguish a green fluorescence pattern from that observed in sperm incubated with PKH67‐labeled epEVs, as shown in Figure 3. In addition, sperm may exhibit intrinsic autofluorescence resulting from the presence of endogenous fluorophores, such as NADH and FAD (Kusari et al. 2025), which may also contribute to the fluorescence observed in the control group.

Interestingly, the interaction between epididymosomes and sperm apparently occurs specifically in the acrosome and midpiece regions, which supports the hypothesis that this regional binding might associated with acrosome reaction and thus with the ability of sperm to fuse with the oocyte. Meanwhile, epididymosomes that bind to the midpiece may affect mitochondrial activity and thus sperm motility. However, it is not exactly established how epididymosomes interact with sperm. Recent evidence showed that this interaction is mediated through fusion mechanisms dependent on the integrity of lipid rafts, that are specialized plasma membrane domains (Sullivan et al. 2007; Zhou et al. 2019), concentrated in the acrosome and flagellar regions of sperm (Shadan et al. 2004; Travis et al. 2001). On the other hand, Zhou et al. (2019) have described that the fusion of epididymosomes to sperm could be facilitated by endogenous mechanoenzyme Dynamin1 localized on the peri‐ and post‐acrosomal region. Since most of the observed binding were not in the post‐acrosomal region in this study, its open new opportunities to study if this interaction is changed by the period of interaction, conditions such as temperature, and other factors that may influence and favor a determined type of sperm‐epididymosomes interaction.

Interesting, when performing seminal plasma EVs incubation with sperm for 1 h, the interaction is observed in the apical region of the sperm head (Gavrilov et al. 2025; Hassani et al. 2025) and in the midpiece (Gavrilov et al. 2025; Lange‐Consiglio et al. 2022) in both bovine and human sperm. In addition, oviductal EVs have been reported to interact with sperm, mainly in the head and midpiece regions in mice (Al‐Dossary et al. 2015), cats (Ferraz et al. 2019), and pigs (Alcântara‐Neto et al. 2020). Thus, these findings indicate that the interaction between EVs and sperm shows specificity, suggesting a precise regulation of this communication. However, none of these evidence was based on incubation of EVs with sperm during cryopreservation, which can change the pattern of interaction. In this study, assessing plasma membrane integrity of sperm from one bull, 27% of sperm that presented plasma membrane integrity visually interacted with epEVs, while 14% of sperm that presented plasma membrane damage visually interacted with epEVs (Supplementary Figure S1). However, further studies investigating the pattern of interactions are urgently needed to better understand the mechanisms involved on epididymosomes communication with sperm.

Once confirmed the interaction between epididymosomes and sperm during cryopreservation, post‐thaw evaluation of sperm morpho‐functional characteristics was performed in samples cryopreserved without (Control group) or with epididymosomes (epEVs group). In that regard, the percentage of post‐thaw sperm with morphological defects showed no difference between groups, as observed for sperm vigor. Regarding subjective motility and motility parameters by computer assisted analysis, sperm from epEVs group showed higher values compared to sperm from Control group. Thus, most of sperm from the epEVs group showed movement, and interestingly, this movement was progressive and fast. On the same way, seminal plasma EVs from normozoospermic men incubated with sperm from teratozoospermic men (Gavrilov et al. 2025) and from oligoastheno‐teratozoospermic men (Hassani et al. 2025), increased the total and progressive motility. Tanrıkulu et al. (2025) found that EVs from stem cells compared to EVs from seminal plasma resulted in higher post‐thaw sperm velocity in canine sperm. Finally, Shamsi et al. (2024) described that post‐thaw sperm cryopreserved with seminal plasma EVs showed higher total and progressive motility, as well as ALH, BCF, VAP, VCL, and VSL parameters. Interestingly, the subjective motility evaluated before cryopreservation in the present study was similar between the groups showing a beneficial effect of the epididymosomes during cryopreservation.

Usually, samples with higher motility present higher fertility ability (Fernández‐López et al. 2022; Gillan et al. 2008) since sperm motility is fundamental to sperm reach the site of fertilization and cross the cumulus cells to reach the oocyte zona pellucida. In fact, sperm motility is impaired by cryopreservation, and this is directly related with the damage to sperm plasma membrane during cryopreservation process (Celeghini et al. 2008). However, in addition to promote protection of membranes, epididymosomes may deliver proteins (Eickhoff et al. 2001; Frenette et al. 2003; Griffiths et al. 2008), lipids (Schwarz et al. 2013), and miRNAs (Ding et al. 2021) that favor motility function. In addition, since cryopreservation is known to induce capacitation‐like changes in sperm (Bailey et al. 2000), while parameters related to hyperactivation (i.e., ALH and VCL) (Stauss et al. 1995; Suárez and Osman 1987) were higher for sperm treated with epididymosomes, percentage of hyperactivated sperm, that associate these different parameters, was similar between the groups. Thus, sperm motility parameters indicated that sperm cryopreserved with epididymosomes were protected to damage during cryopreservation probably by protecting plasma membrane or also delivering molecules to sperm. Also, these results suggest a protection to pass through “cryo‐capacitation.”

Regarding sperm membrane integrity, the epEVs group showed a higher percentage of sperm with intact plasma membranes when assessed by flow cytometry, but not by fluorescence microscopy. In this case, the evaluation process of each technique must be considered, as flow cytometry enables the analysis of a much larger sperm population (20,000 sperm cells) compared with fluorescence microscopy, in which only 200 cells were evaluated. Therefore, flow cytometry is expected to provide a more robust assessment of plasma membrane integrity compared to fluorescence microscopy. Recent studies have reported a higher presence of viable sperm in bovine and human samples, reflected by intact plasma membranes (Kowalczyk and Kordan 2024; Shamsi et al. 2024) and high mitochondrial membrane potential (Gavrilov et al. 2025; Kowalczyk and Kordan 2024), when cryopreserved in the presence of seminal plasma‐derived EVs, as well as increased plasma membrane integrity in canine sperm cryopreserved with EVs derived from adipose tissue mesenchymal stem cells (Tanrıkulu et al. 2025). In the present study, although no statistical difference was found for mitochondrial membrane potential, an increase in PIAIA‐positive cells was observed by fluorescence microscopy, along with higher mitochondrial membrane potential detected by flow cytometry in the spEVs group. These results may be directly related to the maintenance of sperm motility and fertility, as plasma membrane integrity ensures regulated ion and molecule fluxes, preserves cellular homeostasis, and supports ATP production through improved mitochondrial preservation (Góngora et al. 2025). Considering that, during cryopreservation, antioxidant enzymes in sperm undergo denaturation and degradation, leading to increased production of reactive oxygen species (ROS) and compromising post‐thaw motility and membrane integrity (Mislei et al. 2020), the intrinsic molecular cargo of epididymosomes may enhance sperm cryotolerance, reducing ROS‐related damage and helping to maintain sperm viability during cryopreservation. In addition, *cauda* epididymosomes are rich in sphingomyelin and arachidonic acid (Rejraji et al. 2006), lipid fractions present in the sperm membrane (Schiller et al. 2003; Shan et al. 2021). Since, during cryopreservation, sperm plasma membrane phospholipids undergo structural remodeling, with changes from a liquid to a gel state, resulting in lipid phase separation and potential loss of integrity (Quinn 1989), the addition of epididymosomes may promote greater preservation of the plasma membrane and consequently improved post‐thaw sperm viability.

After sperm post‐thaw, molecular analyses of the miRNA levels of cryopreserved sperm from the Control and epEV groups revealed that the epEVs group exhibited a greater number of up‐regulated (5 miRNAs) and exclusive (23 miRNAs) miRNAs compared to the Control group. Among these total of 28 miRNAs found in our study, miR‐106a, miR‐127, miR‐134, miR‐210, miR‐330, and miR‐677 have already been reported as enclosed in mouse *cauda* epididymosomes (Reilly et al. 2016; Sharma et al. 2016), and let‐7b, let‐7i, miR‐10a, miR‐10b, miR‐15b, miR‐16a, miR‐34a, miR‐140, miR‐200a, miR‐210 and miR‐423‐3p have been described in *cauda* epididymosomes from both mouse and cattle (Belleannée et al. 2013; Reilly et al. 2016; Sharma et al. 2016). It is important to consider that epEVs potentially transfer other types of molecules to sperm as lipids and proteins, that are may involved in sperm viability and fertility potential, as well as other types of RNAs, including other classes of sncRNAs with a potential role in sperm contribution to development (Chen et al. 2021; Conine et al. 2018). However, since miRNAs are well recognized in terms of sperm contribution to development, which includes effects on sperm fertility potential and early embryonic development (Alves et al. 2019; Isacson et al. 2025; W.‐M. Liu et al. 2012; Pinto et al. 2025; Trigg and Conine 2024; Wang et al. 2017; Yuan et al. 2015), the investigation of miRNAs could shed light on essential information on the role of epididymosomes in cryopreservation.

Although it is established that sperm are transcriptionally inert and that there is no confirmatory evidence that miRNAs act directly on mature sperm cells, it is known that sperm carry distinct transcripts in the different compartments of the epididymis, such as miRNAs and mRNAs (Belleannée et al. 2013; Trigg et al. 2025) and some studies report that these transcripts may vary according to sperm motility quality (Capra et al. 2017; Turri et al. 2021). Ding et al. (2021) reported that the presence of miR‐222 in seminal plasma EVs from pigs contributes to improve sperm motility. In their study, EVs were electroporated with a miR‐222 mimic or inhibitor and then incubated with sperm. The results showed that treatment with the miR‐222 mimic significantly increased sperm motility compared to the inhibitor‐treated group or that in Control sperm. Thus, even though this miRNA was not differentially expressed between groups in the present study, it is speculated that the miRNA content of EVs may regulate the maintenance of sperm viability. In the present study, when performing gene‐target enrichment analysis of the most highly detected and exclusive miRNAs in the epEVs group, we observed biological functions related to intracellular pH regulation, regulation of phosphorylation, and protein transport, which are important for supporting male gamete motility (Carr and Acott 1989; Ferreira et al. 2022; Vijayaraghavan et al. 1997), as well as signaling pathways such as Ras and Wnt, which have also been associated with sperm viability (Koch et al. 2015; NagDas et al. 2002; Zhao et al. 2024).

Moreover, a miRNA (miR‐345‐3p) was found to be highly detected in the Control group compared to the epEVs group. This miRNA has previously been identified as a component of soy lecithin–based extenders (Capra et al. 2024). Interestingly, a previous study of our research group from de Almeida et al. (2024) reported that particles present in such extenders decrease according to the period of incubation with sperm during cryopreservation, suggesting that these particles are incorporated by sperm cells. Therefore, it can be hypothesized that, during cryopreservation in the presence of epididymosomes, sperm exhibit greater interaction with epEVs and, consequently, reduced uptake of molecules originating from the extender. In the Control group, however, this competition would not occur, resulting in greater incorporation of components from the soy lecithin–based extender, such as miR‐345‐3p, which would explain its higher relative expression compared to the epEVs group. Nonetheless, it is important to note that the present study did not investigate the mechanisms involved in the transfer of these molecules to sperm, and further studies are necessary to confirm this possible phenomenon.

Sperm molecular content of miRNAs can be delivered at the moment of fertilization and support early embryonic development, as shown for miR‐449b in cattle (Wang et al. 2017) and miR‐34c in mice (W.‐M. Liu et al. 2012). Furthermore, it has been reported that epididymosomes derived from epididymal cells co‐incubated with sperm modulate the sperm miRNA molecular signature and, consequently, influence offspring traits (Chan et al. 2020). Yin et al. (2025) demonstrated that injecting paternal sperm miRNAs from exercised mice into normal zygotes reproduced exercise‐induced phenotypes in the offspring. Therefore, paternal miRNAs function as epigenetic messengers that not only support embryonic development but also transmit traits to the progeny. In this context, in the present study, the up‐regulated and exclusive miRNAs identified in the group of sperm cryopreserved with epididymosomes exhibited biological functions, based on gene‐target enrichment analysis, related to cell differentiation and embryonic development in the uterus. These functions may be associated with preimplantation embryonic development and subsequent embryo–endometrium interactions during early gestation. The Wnt signaling pathway was also identified, which plays an essential role in cell proliferation and migration, maintenance of stem‐cell pluripotency, and preservation of genetic stability. This pathway is fundamental during the stages of embryonic development (Huelsken et al. 2000; Na et al. 2007). Sidrat et al. (2020) reported that induction of peroxisome proliferator‐activated receptor delta (PPARδ) expression through stimulation of the Wnt/β‐catenin pathway increased cell proliferation and fatty‐acid oxidation metabolism, thereby improving bovine blastocyst development and hatched. Regarding Ras protein signal transduction, these proteins are small GTPases that, when activated, transmit signals from membrane receptors (such as tyrosine kinase receptors) to intracellular effectors, triggering signaling cascades that regulate cell proliferation, survival, differentiation, and motility (Margolis and Skolnik 1994). Lu et al. (2008) highlighted that Ras activation can redirect embryonic stem cells toward extraembryonic trophoblastic lineages and is involved in the Ras–MAPK (mitogen‐activated protein kinase) signaling pathway, supporting trophectoderm formation in mouse embryos. These findings regarding the biological effects of miRNAs are consistent with the results observed in the present *in vitro* embryo production (IVP), in which, although no differences were detected in first cleavage, cleavage, or blastocyst rates, a reduction in early blastocysts and an increase in hatched blastocysts were observed in the epEVs group compared with the Control group. However, the biological functions identified were inferred from enrichment analyses and were not experimentally evaluated in the present study. Therefore, further investigations are required to validate their involvement in the mechanisms associated with the observed effects.

Finally, the blastocyst's ability to hatch is commonly used as an indicator of embryo quality and viability (Bridi et al. 2021; Rodriguez Villamil et al. 2012; Sun et al. 2024). It has been shown that EVs can transport molecules, such as non‐coding RNAs, which play an important role in blastocyst hatched, including transfer RNA‐derived small RNAs (tsRNAs) (Fan et al. 2024) and miRNAs (Pavani et al. 2022). Furthermore, circular RNAs derived from blastocysts can bind to tsRNAs and miRNAs secreted by these EVs, enhancing the hatched rate (Fan et al. 2024). Although these studies did not specifically focus on epididymosomes, based on the present results, it is observed that the Control group showed a lower proportion of hatched blastocysts compared to the epEVs group. Moreover, there was no difference in the blastocyst cell number between the groups. These findings suggest that the higher hatched rate observed in the epEVs group is not related to cell proliferation, but rather to other factors, such as miRNA molecules, which may modulate hatched ability. However, this hypothesis needs further investigation.

Based on the interaction between sperm and EVs and their contribution to early embryonic development, studies have shown that incubating bovine sperm with seminal plasma–derived EVs results in higher blastocyst rates (Lange‐Consiglio et al. 2022). Similar findings have been reported in felines, where incubating epididymal EVs from normospermic cats with sperm from teratospermic cats also increased blastocyst rates (Sosnicki et al. 2025). Despite these advances, no study to date has investigated the fertilizing potential of sperm in the context of *in vitro* embryo production using sperm obtained from batches cryopreserved with EVs. This gap is particularly relevant considering that, during cryopreservation, sperm suffer damage that compromises motility, membrane integrity, and molecular content (Castro et al. 2016; Ribas‐Maynou et al. 2024; Watson 2000). To minimize these effects, various protocols have been developed to preserve sperm quality post‐thaw (Hezavehei et al. 2018). In this context, the use of extenders containing penetrating cryoprotectants (such as glycerol) and non‐penetrating cryoprotectants (such as soybean lecithin), combined with the “equilibrium” time (Leite et al. 2010; van Wagtendonk‐de Leeuw et al. 2000), is a widely adopted strategy to optimize cryopreservation. However, although these efforts mainly focus on improving post‐thaw sperm morpho‐functional quality, the application of EVs during cryopreservation may offer a complementary approach by helping maintain the viability of sperm molecular content and, consequently, increasing sperm fertilizing potential.

## 5 Conclusion

In conclusion, we demonstrated that sperm interact with epididymosomes during cryopreservation. Sperm exposed to epididymosomes during cryopreservation display higher sperm post‐thaw motility and plasma membrane integrity in addition to sperm‐epEVs interaction reprograms the sperm miRNA profile. Finally, cryopreservation of sperm with epididymosomes increases embryo developmental potential, as indicated by hatched rates. The findings of this study provide insights for improving post‐thaw sperm quality and open avenues to comprehension of the factors that guide sperm fertility potential and paternal effect to development.

## Author Contributions

**Laura Gabrielli Haupenthal:** conceptualization, writing – original draft, methodology, investigation. **Maria Alice de Almeida:** methodology, investigation, writing – review and editing. **Cibele Maria Prado:** methodology, investigation, writing – review and editing. **Amanda Nespolo Silva:** methodology, investigation, writing – review and editing. **Gabriela Melendes Schneider:** methodology, investigation, writing – review and editing. **Paola Maria da Silva Rosa:** methodology, investigation, writing – review and editing. **Flávio Vieira Meirelles:** funding acquisition, methodology, writing – review and editing. **Juliano Coelho da Silveira:** methodology, funding acquisition, writing – review and editing. **Felipe Perecin:** methodology, funding acquisition, writing – review and editing. **Maíra Bianchi Rodrigues Alves:** conceptualization, data curation, supervision, methodology, investigation, writing – original draft.

## Conflicts of Interest

The authors declare that there is a patent application requested (BR 10 2023 020354‐0) related to this study.

## Supporting information


Supporting File 1

Supporting File 2

Supporting File 3

Supporting File 4

## Data Availability

The data that support the findings of this study are available from the corresponding author upon reasonable request.

## References

[mrd70138-bib-0001] Colégio Brasileiro Reprodução Animal (CBRA) . 2013. Manual Para Exame Andrológico E Avaliação De Sêmen Animal. 3rd ed.

[mrd70138-bib-0002] Associação Brasileira De Inseminação Artificial . 2025. *Index ASBIA: Relatório trimestral de comercialização de sêmen*.

[mrd70138-bib-0003] Alcântara‐Neto, A. S. , L. Schmaltz , E. Caldas , M.‐C. Blache , P. Mermillod , and C. Almiñana . 2020. “Porcine Oviductal Extracellular Vesicles Interact With Gametes and Regulate Sperm Motility and Survival.” Theriogenology 155: 240–255. 10.1016/j.theriogenology.2020.05.043. 32791377

[mrd70138-bib-0004] Al‐Dossary, A. A. , P. Bathala , J. L. Caplan , and P. A. Martin‐DeLeon . 2015. “Oviductosome‐Sperm Membrane Interaction in Cargo Delivery.” Journal of Biological Chemistry 290, no. 29: 17710–17723. 10.1074/jbc.M114.633156. 26023236 PMC4505020

[mrd70138-bib-0005] de Almeida, M. A. , L. G. Haupenthal , and A. N. Silva , et al. 2024. “A Longer Period of Epididymal Sperm Interaction With Extender Components During Cryopreservation Improves Sperm Quality, Decreases the Size of Sperm Distal Cytoplasmic Droplets, and Changes the Number of Nanoparticles in the Extender.” Cryobiology 115: 104901. 10.1016/j.cryobiol.2024.104901. 38754687

[mrd70138-bib-0006] Alves, M. B. R. , M. A. A. de Almeida , A. B. B. Moura , et al. 2026. “In Vitro Sperm–Epididymosomes Interaction Immediately Before Fertilization Changes Sperm Fertility Potential.” Andrology. 10.1111/andr.70267. PMC13432530 42216502

[mrd70138-bib-0007] Alves, M. B. R. , R. P. Arruda , L. Batissaco , et al. 2021. “Changes in miRNA Levels of Sperm and Small Extracellular Vesicles of Seminal Plasma are Associated With Transient Scrotal Heat Stress in Bulls.” Theriogenology 161: 26–40. 10.1016/j.theriogenology.2020.11.015. 33278692

[mrd70138-bib-0008] Alves, M. B. R. , R. P. de Arruda , and T. H. C. De Bem , et al. 2019. “Sperm‐Borne miR‐216b Modulates Cell Proliferation During Early Embryo Development via K‐RAS.” Scientific Reports 9, no. 1: 10358. 10.1038/s41598-019-46775-8. 31316130 PMC6637201

[mrd70138-bib-0009] Alves, M. B. R. , E. C. C. Celeghini , and C. Belleannée . 2020. “From Sperm Motility to Sperm‐Borne microRNA Signatures: New Approaches to Predict Male Fertility Potential.” Frontiers in Cell and Developmental Biology 8: 791. 10.3389/fcell.2020.00791. 32974342 PMC7471662

[mrd70138-bib-0010] Bailey, J. L. , J.‐F. Blodeau , and N. Cormier . 2000. “Semen Cryopreservation in Domestic Animals: A Damaging and Capacitating Phenomenon Minireview.” Journal of Andrology 21, no. 1: 1–7. 10.1002/j.1939-4640.2000.tb03268.x. 10670514

[mrd70138-bib-0011] Barth, A. , and R. Oko . 1989. Abnormal Morphology of Bovine Spermatozoa. Iowa State University Press.

[mrd70138-bib-0012] Batra, V. , H. L. Morgan , and K. K. Choi , et al. 2025. “Male Reproductive Tract Extracellular Vesicles Display Region‐Specific Heterogeneity in Mice.” Reproduction 170, no. 1: e250009. 10.1530/REP-25-0009. 40424048 PMC12152732

[mrd70138-bib-0013] Belleannée, C. , É. Calvo , J. Caballero , and R. Sullivan . 2013. “Epididymosomes Convey Different Repertoires of microRNAs Throughout the Bovine Epididymis.” Biology of Reproduction 89, no. 2: 30. 10.1095/biolreprod.113.110486. 23803555

[mrd70138-bib-0014] Bridi, A. , G. M. Andrade , M. del Collado , et al. 2021. “Small Extracellular Vesicles Derived From In Vivo‐ or In Vitro‐Produced Bovine Blastocysts Have Different miRNAs Profiles—Implications for Embryo‐Maternal Recognition.” Molecular Reproduction and Development 88, no. 9: 628–643. 10.1002/mrd.23527. 34402123

[mrd70138-bib-0015] Caballero, J. N. , G. Frenette , C. Belleannée , and R. Sullivan . 2013. “CD9‐Positive Microvesicles Mediate the Transfer of Molecules to Bovine Spermatozoa During Epididymal Maturation.” PLoS One 8, no. 6: e65364. 10.1371/journal.pone.0065364. 23785420 PMC3681974

[mrd70138-bib-0016] Capra, E. , R. Frigerio , and B. Lazzari , et al. 2024. “Effect of Cryopreservation and Semen Extender on Extracellular Vesicles Isolated From Bull Semen.” Frontiers in Veterinary Science 11: 1437410. 10.3389/fvets.2024.1437410. 39139604 PMC11321215

[mrd70138-bib-0017] Capra, E. , F. Turri , B. Lazzari , et al. 2017. “Small RNA Sequencing of Cryopreserved Semen From Single Bull Revealed Altered miRNAs and piRNAs Expression Between High‐ and Low‐Motile Sperm Populations.” BMC Genomics 18, no. 1: 14. 10.1186/s12864-016-3394-7. 28052756 PMC5209821

[mrd70138-bib-0018] Carr, D. W. , and T. S. Acott . 1989. “Intracellular pH Regulates Bovine Sperm Motility and Protein Phosphorylation1.” Biology of Reproduction 41, no. 5: 907–920. 10.1095/biolreprod41.5.907. 2624855

[mrd70138-bib-0019] Castro, L. S. , T. R. S. Hamilton , C. M. Mendes , et al. 2016. “Sperm Cryodamage Occurs After Rapid Freezing Phase: Flow Cytometry Approach and Antioxidant Enzymes Activity at Different Stages of Cryopreservation.” Journal of Animal Science and Biotechnology 7, no. 1: 17. 10.1186/s40104-016-0076-x. 26949533 PMC4779270

[mrd70138-bib-0020] Celeghini, E. C. C. , R. P. de Arruda , A. F. C. de Andrade , J. Nascimento , C. F. Raphael , and P. H. M. Rodrigues . 2008. “Effects That Bovine Sperm Cryopreservation Using Two Different Extenders Has on Sperm Membranes and Chromatin.” Animal Reproduction Science 104, no. 2–4: 119–131. 10.1016/j.anireprosci.2007.02.001. 17368970

[mrd70138-bib-0021] Chan, J. C. , C. P. Morgan , N. Adrian Leu , et al. 2020. “Reproductive Tract Extracellular Vesicles are Sufficient to Transmit Intergenerational Stress and Program Neurodevelopment.” Nature Communications 11, no. 1: 1499. 10.1038/s41467-020-15305-w. PMC7083921 32198406

[mrd70138-bib-0022] Chen, X. , Q. Sun , Y. Zheng , et al. 2021. “Human Sperm tsRNA as Potential Biomarker and Therapy Target for Male Fertility.” Reproduction 161, no. 2: 111–122. 10.1530/REP-20-0415. 33434159

[mrd70138-bib-0023] Conine, C. C. , F. Sun , L. Song , J. A. Rivera‐Pérez , and O. J. Rando . 2018. “Small RNAs Gained During Epididymal Transit of Sperm Are Essential for Embryonic Development in Mice.” Developmental Cell 46, no. 4: 470–480.e3. 10.1016/j.devcel.2018.06.024. 30057276 PMC6103825

[mrd70138-bib-0024] Ding, Y. , N. Ding , Y. Zhang , et al. 2021. “MicroRNA‐222 Transferred From Semen Extracellular Vesicles Inhibits Sperm Apoptosis by Targeting BCL2L11.” Frontiers in Cell and Developmental Biology 9. 10.3389/fcell.2021.736864. PMC8607813 34820370

[mrd70138-bib-0025] Eickhoff, R. , B. Wilhelm , and H. Renneberg , et al. 2001. “Purification and Characterization of Macrophage Migration Inhibitory Factor as a Secretory Protein From Rat Epididymis: Evidences for Alternative Release and Transfer to Spermatozoa.” Molecular Medicine 7, no. 1: 27–35. 11474125 PMC1949991

[mrd70138-bib-0026] Ezzati, M. , D. Shanehbandi , B. Bahramzadeh , K. Hamdi , and M. Pashaiasl . 2021. “Investigation of Molecular Cryopreservation, Fertility Potential and microRNA‐Mediated Apoptosis in Oligoasthenoteratozoospermia Men.” Cell and Tissue Banking 22, no. 1: 123–135. 10.1007/s10561-020-09872-x. 33057898

[mrd70138-bib-0027] Fan, Y. , K. C. Pavani , B. J. G. Broeckx , K. Smits , A. Van Soom , and L. Peelman . 2024. “Circular RNAs From Bovine Blastocysts Can Interact With miRNAs/tsRNAs From Embryonic Extracellular Vesicles and Regulate Hatching.” International Journal of Biological Macromolecules 277: 134018. 10.1016/j.ijbiomac.2024.134018. 39032885

[mrd70138-bib-0028] Fan, Y. , K. C. Pavani , K. Smits , A. Van Soom , and L. Peelman . 2024. “tRNAGlu‐Derived Fragments From Embryonic Extracellular Vesicles Modulate Bovine Embryo Hatching.” Journal of Animal Science and Biotechnology 15, no. 1: 23. 10.1186/s40104-024-00997-7. 38424649 PMC10905895

[mrd70138-bib-0029] Fernández‐López, P. , J. Garriga , I. Casas , M. Yeste , and F. Bartumeus . 2022. “Predicting Fertility From Sperm Motility Landscapes.” Communications Biology 5, no. 1: 1015. 10.1038/s42003-022-03954-0. 36171267 PMC9519750

[mrd70138-bib-0030] Ferraz, M. A. M. M. , A. Carothers , R. Dahal , M. J. Noonan , and N. Songsasen . 2019. “Oviductal Extracellular Vesicles Interact With the Spermatozoon's Head and Mid‐Piece and Improves Its Motility and Fertilizing Ability in the Domestic Cat.” Scientific Reports 9, no. 1: 9484. 10.1038/s41598-019-45857-x. 31263184 PMC6603010

[mrd70138-bib-0031] Ferreira, A. F. , J. Santiago , J. V. Silva , P. F. Oliveira , and M. Fardilha . 2022. “PP1, PP2A and PP2B Interplay in the Regulation of Sperm Motility: Lessons From Protein Phosphatase Inhibitors.” International Journal of Molecular Sciences 23, no. 23: 15235. 10.3390/ijms232315235. 36499559 PMC9737803

[mrd70138-bib-0032] Frenette, G. , J. Girouard , O. D'Amours , N. Allard , L. Tessier , and R. Sullivan . 2010. “Characterization of Two Distinct Populations of Epididymosomes Collected in the Intraluminal Compartment of the Bovine Cauda Epididymis.” Biology of Reproduction 83, no. 3: 473–480. 10.1095/biolreprod.109.082438. 20554923

[mrd70138-bib-0033] Frenette, G. , C. Lessard , E. Madore , M. A. Fortier , and R. Sullivan . 2003. “Aldose Reductase and Macrophage Migration Inhibitory Factor Are Associated With Epididymosomes and Spermatozoa in the Bovine Epididymis1.” Biology of Reproduction 69, no. 5: 1586–1592. 10.1095/biolreprod.103.019216. 12826572

[mrd70138-bib-0034] Frenette, G. , and R. Sullivan . 2001. “Prostasome‐Like Particles Are Involved in the Transfer of P25b From the Bovine Epididymal Fluid to the Sperm Surface.” Molecular Reproduction and Development 59, no. 1: 115–121. 10.1002/mrd.1013. 11335953

[mrd70138-bib-0035] Gavrilov, M. , N. Makarova , and A. Sysoeva , et al. 2025. “Changes in Cryotolerance of Spermatozoa in Men With Teratozoospermia Under the Influence of Extracellular Vesicles From Donor Seminal Plasma Isolated by Depth Filtration.” Life 15, no. 9: 1436. 10.3390/life15091436. 41010378 PMC12471674

[mrd70138-bib-0036] Gillan, L. , T. Kroetsch , W. M. Chis Maxwell , and G. Evans . 2008. “Assessment of In Vitro Sperm Characteristics in Relation to Fertility in Dairy Bulls.” Animal Reproduction Science 103, no. 3–4: 201–214. 10.1016/j.anireprosci.2006.12.010. 17208395

[mrd70138-bib-0037] Girouard, J. , G. Frenette , and R. Sullivan . 2011. “Comparative Proteome and Lipid Profiles of Bovine Epididymosomes Collected in the Intraluminal Compartment of the Caput and Cauda Epididymidis.” International Journal of Andrology 34, no. 5pt2: e475–e486. 10.1111/j.1365-2605.2011.01203.x. 21875428

[mrd70138-bib-0038] Góngora, A. , S. Johnston , P. Contreras , C. López‐Fernández , and J. Gosálvez . 2025. “The Nexus Between Sperm Membrane Integrity, Sperm Motility, and DNA Fragmentation.” Membranes 15, no. 4: 109. 10.3390/membranes15040109. 40277979 PMC12028733

[mrd70138-bib-0039] Griffiths, G. S. , D. S. Galileo , K. Reese , and P. A. Martin‐DeLeon . 2008. “Investigating the Role of Murine Epididymosomes and Uterosomes in GPI‐Linked Protein Transfer to Sperm Using SPAM1 as a Model.” Molecular Reproduction and Development 75, no. 11: 1627–1636. 10.1002/mrd.20907. 18384048

[mrd70138-bib-0040] Hassani, R. , H. R. Asgari , M. Koruji , Z. Zandieh , and Z. Nazmara . 2025. “Normozoospermic Seminal Fluid Extracellular Vesicles Could Ameliorate Adverse Effects of Freeze–Thaw in Oligoasthenoteratospermia Men.” Journal Of Assisted Reproduction And Genetics 42, no. 11: 3979–3991. 10.1007/s10815-025-03597-0. 40824515 PMC12640299

[mrd70138-bib-0041] Hezavehei, M. , M. Sharafi , H. M. Kouchesfahani , et al. 2018. “Sperm Cryopreservation: A Review on Current Molecular Cryobiology and Advanced Approaches.” Reproductive BioMedicine Online 37, no. 3: 327–339. 10.1016/j.rbmo.2018.05.012. 30143329

[mrd70138-bib-0042] Huelsken, J. , R. Vogel , V. Brinkmann , B. Erdmann , C. Birchmeier , and W. Birchmeier . 2000. “Requirement for β‐Catenin in Anterior‐Posterior Axis Formation in Mice.” Journal of Cell Biology 148, no. 3: 567–578. 10.1083/jcb.148.3.567. 10662781 PMC2174807

[mrd70138-bib-0043] Isacson, S. , K. Karlsson , S. Zalavary , et al. 2025. “Small RNA in Sperm–Paternal Contributions to Human Embryo Development.” Nature Communications 16, no. 1: 6571. 10.1038/s41467-025-62015-2. PMC12267487 40670377

[mrd70138-bib-0044] Koch, S. , S. P. Acebron , J. Herbst , G. Hatiboglu , and C. Niehrs . 2015. “Post‐Transcriptional Wnt Signaling Governs Epididymal Sperm Maturation.” Cell 163, no. 5: 1225–1236. 10.1016/j.cell.2015.10.029. 26590424

[mrd70138-bib-0045] Kowal, J. , G. Arras , M. Colombo , et al. 2016. “Proteomic Comparison Defines Novel Markers to Characterize Heterogeneous Populations of Extracellular Vesicle Subtypes.” Proceedings of the National Academy of Sciences of the United States of America 113, no. 8: 968–977. 10.1073/pnas.1521230113. PMC4776515 26858453

[mrd70138-bib-0046] Kowalczyk, A. , and W. Kordan . 2024. “Evaluation of the Effectiveness of the Use of Exosomes in the Regulation of the Mitochondrial Membrane Potential of Frozen/Thawed Spermatozoa.” PLoS One 19, no. 7: e0303479. 10.1371/journal.pone.0303479. 38959270 PMC11221688

[mrd70138-bib-0047] Kusari, F. , L. Backova , D. Panek , A. Benda , and Z. Trachtulec . 2025. “Label‐Free Metabolic Fingerprinting of Motile Mammalian Spermatozoa With Subcellular Resolution.” BMC Biology 23, no. 1: 85. 10.1186/s12915-025-02167-1. 40128804 PMC11934609

[mrd70138-bib-0048] Lange‐Consiglio, A. , E. Capra , N. Monferini , et al. 2022. “Extracellular Vesicles From Seminal Plasma to Improve Fertilizing Capacity of Bulls.” Reproduction and Fertility 3, no. 4: 313–327. 10.1530/RAF-22-0037. 36374278 PMC9782411

[mrd70138-bib-0049] Leite, T. G. , V. R. do Vale Filho , R. P. de Arruda , et al. 2010. “Effects of Extender and Equilibration Time on Post‐Thaw Motility and Membrane Integrity of Cryopreserved Gyr Bull Semen Evaluated by CASA and Flow Cytometry.” Animal Reproduction Science 120, no. 1–4: 31–38. 10.1016/j.anireprosci.2010.04.005. 20434857

[mrd70138-bib-0050] Liu, W.‐M. , R. T. K. Pang , P. C. N. Chiu , et al. 2012. “Sperm‐Borne microRNA‐34c is Required for the First Cleavage Division in Mouse.” Proceedings of the National Academy of Sciences 109, no. 2: 490–494. 10.1073/pnas.1110368109. PMC3258645 22203953

[mrd70138-bib-0051] Liu, Y. , C. Liang , Y. Gao , et al. 2019. “Fluoride Interferes With the Sperm Fertilizing Ability via Downregulated SPAM1, ACR, and PRSS21 Expression in Rat Epididymis.” Journal of Agricultural and Food Chemistry 67, no. 18: 5240–5249. 10.1021/acs.jafc.9b01114. 31008594

[mrd70138-bib-0052] Lu, C.‐W. , A. Yabuuchi , L. Chen , S. Viswanathan , K. Kim , and G. Q. Daley . 2008. “Ras‐MAPK Signaling Promotes Trophectoderm Formation From Embryonic Stem Cells and Mouse Embryos.” Nature Genetics 40, no. 7: 921–926. 10.1038/ng.173. 18536715 PMC2690707

[mrd70138-bib-0053] Luo, J. , S. Zhu , Y. Kang , et al. 2024. “Isolation of CD63‐Positive Epididymosomes From Human Semen and Its Application in Improving Sperm Function.” Journal of Extracellular Vesicles 13, no. 10. 10.1002/jev2.70006. PMC11483612 39417597

[mrd70138-bib-0054] Margolis, B. , and E. Y. Skolnik . 1994. “Activation of Ras by Receptor Tyrosine Kinases.” Journal of the American Society of Nephrology 5, no. 6: 1288–1299. 10.1681/ASN.V561288. 7893993

[mrd70138-bib-0055] Mislei, B. , D. Bucci , E. Malama , H. Bollwein , and G. Mari . 2020. “Seasonal Changes in ROS Concentrations and Sperm Quality in Unfrozen and Frozen‐Thawed Stallion Semen.” Theriogenology 144: 89–97. 10.1016/j.theriogenology.2019.12.016. 31927419

[mrd70138-bib-0056] Na, J. , K. Lykke‐Andersen , M. E. Torres Padilla , and M. Zernicka‐Goetz . 2007. “Dishevelled Proteins Regulate Cell Adhesion in Mouse Blastocyst and Serve to Monitor Changes in Wnt Signaling.” Developmental Biology 302, no. 1: 40–49. 10.1016/j.ydbio.2006.08.036. 17005174 PMC3353122

[mrd70138-bib-0057] NagDas, S. K. , V. P. Winfrey , and G. E. Olson . 2002. “Identification of Ras and Its Downstream Signaling Elements and Their Potential Role in Hamster Sperm Motility1.” Biology of Reproduction 67, no. 4: 1058–1066. 10.1095/biolreprod67.4.1058. 12297518

[mrd70138-bib-0058] Nixon, B. , S. J. Stanger , and B. P. Mihalas , et al. 2015. “The microRNA Signature of Mouse Spermatozoa is Substantially Modified During Epididymal Maturation1.” Biology of Reproduction 93, no. 4: 91. 10.1095/biolreprod.115.132209. 26333995

[mrd70138-bib-0059] Pavani, K. C. , T. Meese , and O. B. Pascottini , et al. 2022. “Hatching Is Modulated by microRNA‐378a‐3p Derived From Extracellular Vesicles Secreted by Blastocysts.” Proceedings of the National Academy of Sciences 119, no. 12: e2122708119. 10.1073/pnas.2122708119. PMC8944274 35298333

[mrd70138-bib-0060] Pinto, S. , S. C. Pereira , A. Rocha , A. Barros , M. G. Alves , and P. F. Oliveira . 2025. “Sperm‐Borne miR‐34c‐5p and miR‐191‐3p as Markers for Sperm Motility and Embryo Developmental Competence.” Andrology 13, no. 3: 519–530. 10.1111/andr.13698. 39044679

[mrd70138-bib-0061] Plant, T. , and A. Zeleznik . 2015. “The Epididymis.” In Physiology of Reproduction, 691–771. Oxford: Academic Press.

[mrd70138-bib-0062] Quinn, P. J. 1989. “Principles of Membrane Stability and Phase Behavior Under Extreme Conditions.” Journal of Bioenergetics and Biomembranes 21, no. 1: 3–19. 10.1007/BF00762209. 2651426

[mrd70138-bib-0063] R Villamil, P. , D. Lozano , J. M. Oviedo , F. L. Ongaratto , and G. A. Bó . 2012. “Developmental Rates of In Vivo and In Vitro Produced Bovine Embryos Cryopreserved in Ethylene Glycol‐Based Solutions by Slow Freezing or Solid Surface Vitrification.” Animal Reproduction 9, no. 2: 57–62.

[mrd70138-bib-0064] Rangel, R. B. , A. B. B. Moura , L. G. Haupenthal , et al. 2025. “Soma‐Sperm Communication During the Journey to Fertilization: Addressing Challenges and Opportunities.” Biology of Reproduction 114, no. 4: 1130–1150. 10.1093/biolre/ioaf277. 41459761

[mrd70138-bib-0065] Reilly, J. N. , E. A. McLaughlin , and S. J. Stanger , et al. 2016. “Characterisation of Mouse Epididymosomes Reveals a Complex Profile of microRNAs and a Potential Mechanism for Modification of the Sperm Epigenome.” Scientific Reports 6: 31794. 10.1038/srep31794. 27549865 PMC4994100

[mrd70138-bib-0066] Rejraji, H. , B. Sion , G. Prensier , et al. 2006. “Lipid Remodeling of Murine Epididymosomes and Spermatozoa During Epididymal Maturation1.” Biology of Reproduction 74, no. 6: 1104–1113. 10.1095/biolreprod.105.049304. 16510839

[mrd70138-bib-0067] Ribas‐Maynou, J. , R. Muiño , C. Tamargo , and M. Yeste . 2024. “Cryopreservation of Bovine Sperm Causes Single‐Strand DNA Breaks That Are Localized in the Toroidal Regions of Chromatin.” Journal of Animal Science and Biotechnology 15, no. 1: 140. 10.1186/s40104-024-01099-0. 39394604 PMC11470689

[mrd70138-bib-0068] Ryu, D.‐Y. , W.‐H. Song , W.‐K. Pang , S.‐J. Yoon , M. S. Rahman , and M.‐G. Pang . 2019. “Freezability Biomarkers in Bull Epididymal Spermatozoa.” Scientific Reports 9, no. 1: 12797. 10.1038/s41598-019-49378-5. 31488871 PMC6728342

[mrd70138-bib-0069] Schiller, J. , K. Müller , R. Süß , et al. 2003. “Analysis of the Lipid Composition of Bull Spermatozoa by MALDI‐TOF Mass Spectrometry—A Cautionary Note.” Chemistry and Physics of Lipids 126, no. 1: 85–94. 10.1016/S0009-3084(03)00097-5. 14580713

[mrd70138-bib-0070] Schmittgen, T. D. , and K. J. Livak . 2008. “Analyzing Real‐Time PCR Data by the Comparative CT Method.” Nature Protocols 3, no. 6: 1101–1108. 10.1038/nprot.2008.73. 18546601

[mrd70138-bib-0071] Schwarz, A. , G. Wennemuth , H. Post , T. Brandenburger , G. Aumüller , and B. Wilhelm . 2013. “Vesicular Transfer of Membrane Components to Bovine Epididymal Spermatozoa.” Cell and Tissue Research 353, no. 3: 549–561. 10.1007/s00441-013-1633-7. 23715721

[mrd70138-bib-0072] Shadan, S. , P. S. James , E. A. Howes , and R. Jones . 2004. “Cholesterol Efflux Alters Lipid Raft Stability and Distribution During Capacitation of Boar Spermatozoa1.” Biology of Reproduction 71, no. 1: 253–265. 10.1095/biolreprod.103.026435. 15028630

[mrd70138-bib-0073] Shamsi, R. R. , R. J. Jozani , R. Asadpour , M. Rahbar , and M. Taravat . 2025. “Seminal Plasma‐Derived Exosome Preserves the Quality Parameters of the Post‐Thaw Semen of Bulls With Low Freezeability.” Biopreservation and Biobanking 23: 364–373. 10.1089/bio.2024.0077. 39723439

[mrd70138-bib-0074] Shan, S. , F. Xu , M. Hirschfeld , and B. Brenig . 2021. “Sperm Lipid Markers of Male Fertility in Mammals.” International Journal of Molecular Sciences 22, no. 16: 8767. 10.3390/ijms22168767. 34445473 PMC8395862

[mrd70138-bib-0075] Sharma, U. , C. C. Conine , J. M. Shea , et al. 2016. “Biogenesis and Function of tRNA Fragments During Sperm Maturation and Fertilization in Mammals.” Science 351, no. 6271: 391–396. 10.1126/science.aad6780. 26721685 PMC4888079

[mrd70138-bib-0076] Sidrat, T. , A. A. Khan , and M. Idrees , et al. 2020. “Role of Wnt Signaling During In‐Vitro Bovine Blastocyst Development and Maturation in Synergism With PPARδ Signaling.” Cells 9, no. 4: 923. 10.3390/cells9040923. 32283810 PMC7226827

[mrd70138-bib-0077] da Silveira, J. C. , G. M. Andrade , and M. del Collado , et al. 2017. “Supplementation With Small‐Extracellular Vesicles From Ovarian Follicular Fluid During in Vitro Production Modulates Bovine Embryo Development.” PLoS One 12, no. 6: e0179451. 10.1371/journal.pone.0179451. 28617821 PMC5472319

[mrd70138-bib-0078] Skerget, S. , M. A. Rosenow , K. Petritis , and T. L. Karr . 2015. “Sperm Proteome Maturation in the Mouse Epididymis.” PLoS One 10, no. 11: e0140650. 10.1371/journal.pone.0140650. 26556802 PMC4640836

[mrd70138-bib-0079] Sosnicki, D. M. , A. J. Travis , and P. Comizzoli . 2025. “In Vitro Exposure to Epididymal Extracellular Vesicles From Normospermic Domestic Cats Improves Developmental Potential of Sperm From Teratospermic Cats.” Frontiers in Veterinary Science 12: 1547175. 10.3389/fvets.2025.1547175. 39896847 PMC11783848

[mrd70138-bib-0080] Stauss, C. R. , T. J. Votta , and S. S. Suarez . 1995. “Sperm Motility Hyperactivation Facilitates Penetration of the Hamster Zona Pellucida1.” Biology of Reproduction 53, no. 6: 1280–1285. 10.1095/biolreprod53.6.1280. 8562682

[mrd70138-bib-0081] Stringfellow, D. , and M. Givens . 2010. “A Procedural Guide and General Information for the Use of Embryo Transfer Technology Emphasizing Sanitary Procedures.” In International Embryo Transfer Society (4th ed. Manual of the International Embryo Transfer Society).

[mrd70138-bib-0082] Suárez, S. S. , and R. A. Osman . 1987. “Initiation of Hyperactivated Flagellar Bending in Mouse Sperm Within the Female Reproductive Tract1.” Biology of Reproduction 36, no. 5: 1191–1198. 10.1095/biolreprod36.5.1191. 3620562

[mrd70138-bib-0083] Sullivan, R. , G. Frenette , and J. Girouard . 2007. “Epididymosomes Are Involved in the Acquisition of New Sperm Proteins During Epididymal Transit.” Asian Journal of Andrology 9, no. 4: 483–491. 10.1111/j.1745-7262.2007.00281.x. 17589785

[mrd70138-bib-0084] Sun, L. , Y. Wang , and M. Yang , et al. 2024. “Delayed Blastocyst Formation Reduces the Quality and Hatching Ability of Porcine Parthenogenetic Blastocysts by Increasing DNA Damage, Decreasing Cell Proliferation, and Altering Transcription Factor Expression Patterns.” Journal of Developmental Biology 12, no. 4: 26. 10.3390/jdb12040026. 39449318 PMC11503403

[mrd70138-bib-0085] Tanrıkulu, M. D. , M. Çevi̇k , M. Yüce , P. Neslihan Taşlı , and K. Yıldırım . 2025. “Cryoprotective Effects of Mesenchymal Stem Cell and Seminal Plasma‐Derived Extracellular Vesicles on Canine Sperm.” Theriogenology 244: 117480. 10.1016/j.theriogenology.2025.117480. 40381592

[mrd70138-bib-0086] Théry, C. , K. W. Witwer , and E. Aikawa , et al. 2018. “Minimal Information for Studies of Extracellular Vesicles 2018 (MISEV2018): A Position Statement of the International Society for Extracellular Vesicles and Update of the MISEV2014 Guidelines.” Journal of Extracellular Vesicles 7, no. 1: 1535750. 10.1080/20013078.2018.1535750. 30637094 PMC6322352

[mrd70138-bib-0087] Thimon, V. , G. Frenette , F. Saez , M. Thabet , and R. Sullivan . 2008. “Protein Composition of Human Epididymosomes Collected During Surgical Vasectomy Reversal: A Proteomic and Genomic Approach.” Human Reproduction 23, no. 8: 1698–1707. 10.1093/humrep/den181. 18482993

[mrd70138-bib-0088] Thomas, C. A. , D. L. Garner , J. M. DeJarnette , and C. E. Marshall . 1998. “Effect of Cryopreservation on Bovine Sperm Organelle Function and Viability as Determined by Flow Cytometry1.” Biology of Reproduction 58, no. 3: 786–793. 10.1095/biolreprod58.3.786. 9510967

[mrd70138-bib-0089] Travis, A. J. , T. Merdiushev , L. A. Vargas , et al. 2001. “Expression and Localization of Caveolin‐1, and the Presence of Membrane Rafts, in Mouse and Guinea Pig Spermatozoa.” Developmental Biology 240, no. 2: 599–610. 10.1006/dbio.2001.0475. 11784086

[mrd70138-bib-0090] Trigg, N. A. , and C. C. Conine . 2024. “Epididymal Acquired Sperm microRNAs Modify Post‐Fertilization Embryonic Gene Expression.” Cell Reports 43, no. 9: 114698. 10.1016/j.celrep.2024.114698. 39226174

[mrd70138-bib-0091] Trigg, N. A. , G. S. Lee , A. G. Leach , and C. C. Conine (2025). “Epididymal Extracellular Vesicles Harbor and Convey mRNA to Sperm for Transfer to Zygotes.” 10.1101/2025.08.18.670952. PMC13092975 42003555

[mrd70138-bib-0092] Turri, F. , E. Capra , B. Lazzari , P. Cremonesi , A. Stella , and F. Pizzi . 2021. “A Combined Flow Cytometric Semen Analysis and miRNA Profiling as a Tool to Discriminate Between High‐ and Low‐Fertility Bulls.” Frontiers in Veterinary Science 8: 703101. 10.3389/fvets.2021.703101. 34355036 PMC8329915

[mrd70138-bib-0093] Turri, F. , M. Madeddu , T. Gliozzi , G. Gandini , and F. Pizzi . 2012. “Influence of Recovery Methods and Extenders on Bull Epididymal Spermatozoa Quality.” Reproduction in Domestic Animals 47, no. 5: 712–717. 10.1111/j.1439-0531.2011.01948.x. 22107087

[mrd70138-bib-0094] Vijayaraghavan, S. , K. D. Trautman , S. A. Goueli , and D. W. Carr . 1997. “A Tyrosine‐Phosphorylated 55‐Kilodalton Motility‐Associated Bovine Sperm Protein is Regulated by Cyclic Adenosine 3′,5′‐Monophosphates and Calcium1.” Biology of Reproduction 56, no. 6: 1450–1457. 10.1095/biolreprod56.6.1450. 9166697

[mrd70138-bib-0095] van Wagtendonk‐de Leeuw, A. M. , R. M. Haring , L. M. T. E. Kaal‐Lansbergen , and J. H. G. den Daas . 2000. “Fertility Results Using Bovine Semen Cryopreserved With Extenders Based on Egg Yolk and Soy Bean Extract.” Theriogenology 54, no. 1: 57–67. 10.1016/S0093-691X(00)00324-1. 10990347

[mrd70138-bib-0096] Wang, M. , Y. Gao , and P. Qu , et al. 2017. “Sperm‐Borne miR‐449b Influences Cleavage, Epigenetic Reprogramming and Apoptosis of SCNT Embryos in Bovine.” Scientific Reports 7, no. 1: 13403. 10.1038/s41598-017-13899-8. 29042680 PMC5645405

[mrd70138-bib-0097] Watson, P. F. 2000. “The Causes of Reduced Fertility With Cryopreserved Semen.” Animal Reproduction Science 60–61: 481–492. 10.1016/S0378-4320(00)00099-3. 10844218

[mrd70138-bib-0098] Welsh, J. A. , D. C. I. Goberdhan , and L. O'Driscoll , et al. 2024. “Minimal Information for Studies of Extracellular Vesicles (MISEV2023): From Basic to Advanced Approaches.” Journal of Extracellular Vesicles 13, no. 2: e12404. 10.1002/jev2.12404. 38326288 PMC10850029

[mrd70138-bib-0099] Xu, X. , W. Li , and L. Zhang , et al. 2021. “Effect of Sperm Cryopreservation on miRNA Expression and Early Embryonic Development.” Frontiers in Cell and Developmental Biology 9: 749486. 10.3389/fcell.2021.749486. 35004670 PMC8728010

[mrd70138-bib-0100] Yin, X. , A. Anwar , L. Yan , et al. 2025. “Paternal Exercise Confers Endurance Capacity to Offspring Through Sperm microRNAs.” Cell Metabolism 37, no. 11: 2167–2184.e8. 10.1016/j.cmet.2025.09.003. 41056946

[mrd70138-bib-0101] Yuan, S. , A. Schuster , and C. Tang , et al. 2015. “Sperm‐Borne miRNAs and Endo‐siRNAs Are Important for Fertilization and Preimplantation Embryonic Development.” Development 142: 1646–1655. 10.1242/dev.131755. PMC4760322 26718009

[mrd70138-bib-0102] Zhao, Y. , J. Qin , J. Sun , et al. 2024. “Motility‐Related microRNAs Identified in Pig Seminal Plasma Exosomes by High‐Throughput Small RNA Sequencing.” Theriogenology 215: 351–360. 10.1016/j.theriogenology.2023.11.028. 38150851

[mrd70138-bib-0103] Zhou, W. , S. J. Stanger , A. L. Anderson , et al. 2019. “Mechanisms of Tethering and Cargo Transfer During Epididymosome‐Sperm Interactions.” BMC Biology 17, no. 1: 35. 10.1186/s12915-019-0653-5. 30999907 PMC6474069

