# Supplementary material for: Epididymosome‐Supplemented Extender Induces Changes in Morpho‐Functional Traits and microRNA Levels of Post‐Thaw Sperm and Improves Embryo Developmental Potential
Source: Mol Reprod Dev. 2026 Jul 30;93(8):e70138. doi: 10.1002/mrd.70138 (PMC13420828; doi:10.1002/mrd.70138)

Title

Epididymosome-supplemented extender induces changes in morpho-functional traits and microRNA levels of post-thaw sperm and improves embryo developmental potential

Authors

Laura Gabrielli Haupenthal[1], Maria Alice Almeida[1], Cibele Maria Prado[1], Amanda Nespolo Silva[1], Gabriela Melendes Schneider[1], Paola Maria da Silva Rosa[1], Flávio Vieira Meirelles[1], Juliano Coelho da Silveira[1], Felipe Perecin[1], Maíra Bianchi Rodrigues Alves[2*]

Author's institutional affiliations

[1]Department of Veterinary Medicine, School of Animal Science and Food Engineering, University of São Paulo, Pirassununga, São Paulo, Brazil.

[2]Department of Pathology, Theriogenology and One Healthy, School of Agricultural and Veterinary Sciences, São Paulo State University, Jaboticabal, São Paulo, Brazil.

Supplementary Figures

Supplementary Figure S1. Photomicrograph obtained by epifluorescence microscopy (Thunder Imager 3D Assay®; Leica) at 630× magnification of post-thaw sperm cryopreserved with presence of PKH67® green-labeled epididymosomes (epEVs group) stained for plasma membrane integrity markers. For this purpose, sperm batches from one bull of epEVs group were thawed and $10x10^6$ sperm/mL in Talp-Sperm were stained with Hoechst 33342 (10 μg/mL) and propidium iodide (0.5 mg/mL; P4170) and incubated for 8 minutes at 37°C. After, glasses were mounted with cover glass, and images were acquired from different fields using fluorescence microscopy (Thunder Imager 3D Assay®; Leica) at 630× magnification in which 180 sperm were classified to determine the percentage of sperm with integrity or damage plasma membranes that interacted with epEVs. For this analysis, the sperm samples from the bull that showed the highest sperm–epEVs interaction was used. White arrows and ***a’***, indicate sperm exhibiting plasma membrane integrity and interacting with epEVs, whereas red arrows and ***b’,*** indicate sperm exhibiting plasma membrane damage and interacting with epEVs. Scale bar: 10 μm.

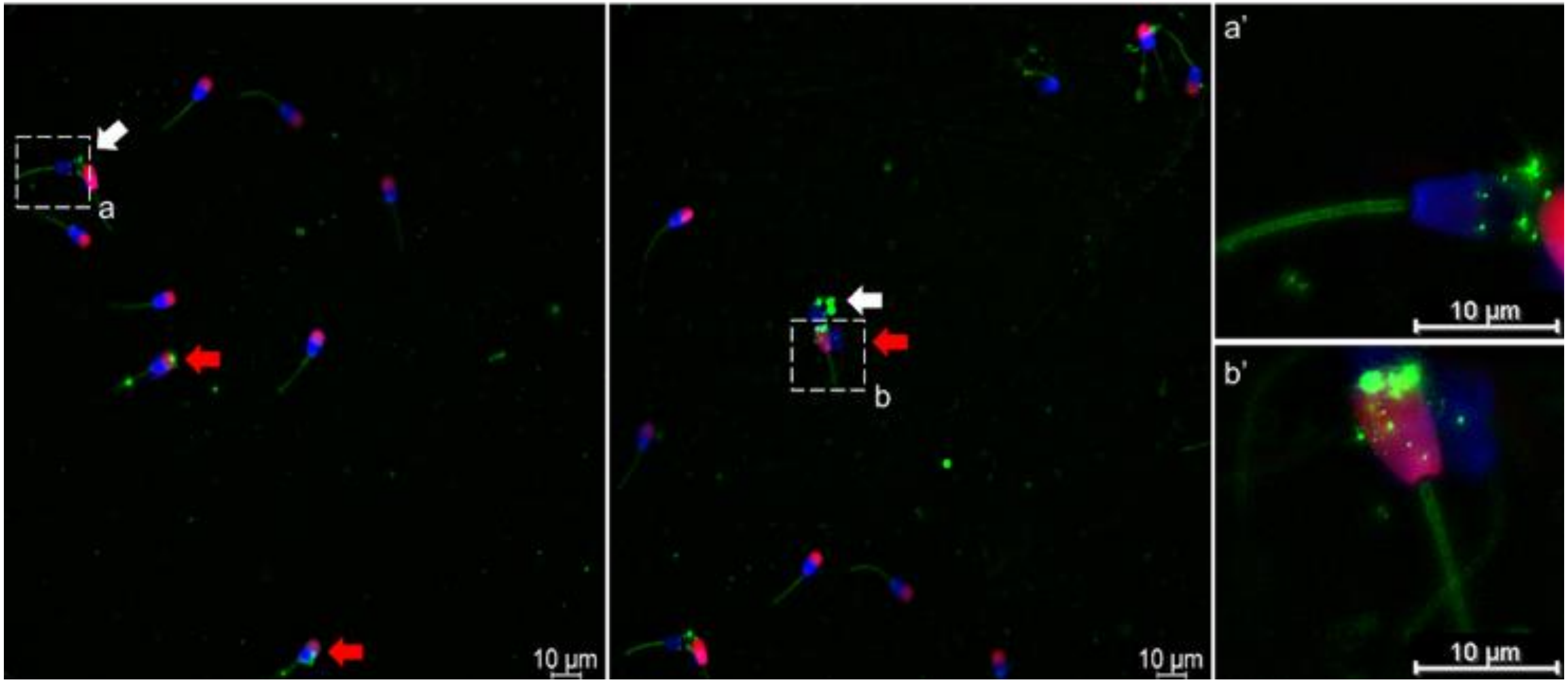
a
10 µm
b
10 µm
a'
10 µm
b'
10 µm

Supplementary Figure S2. Image of blastocysts generated in a routine using sperm from the Control and epEVs groups. Black arrows indicate hatched blastocysts.

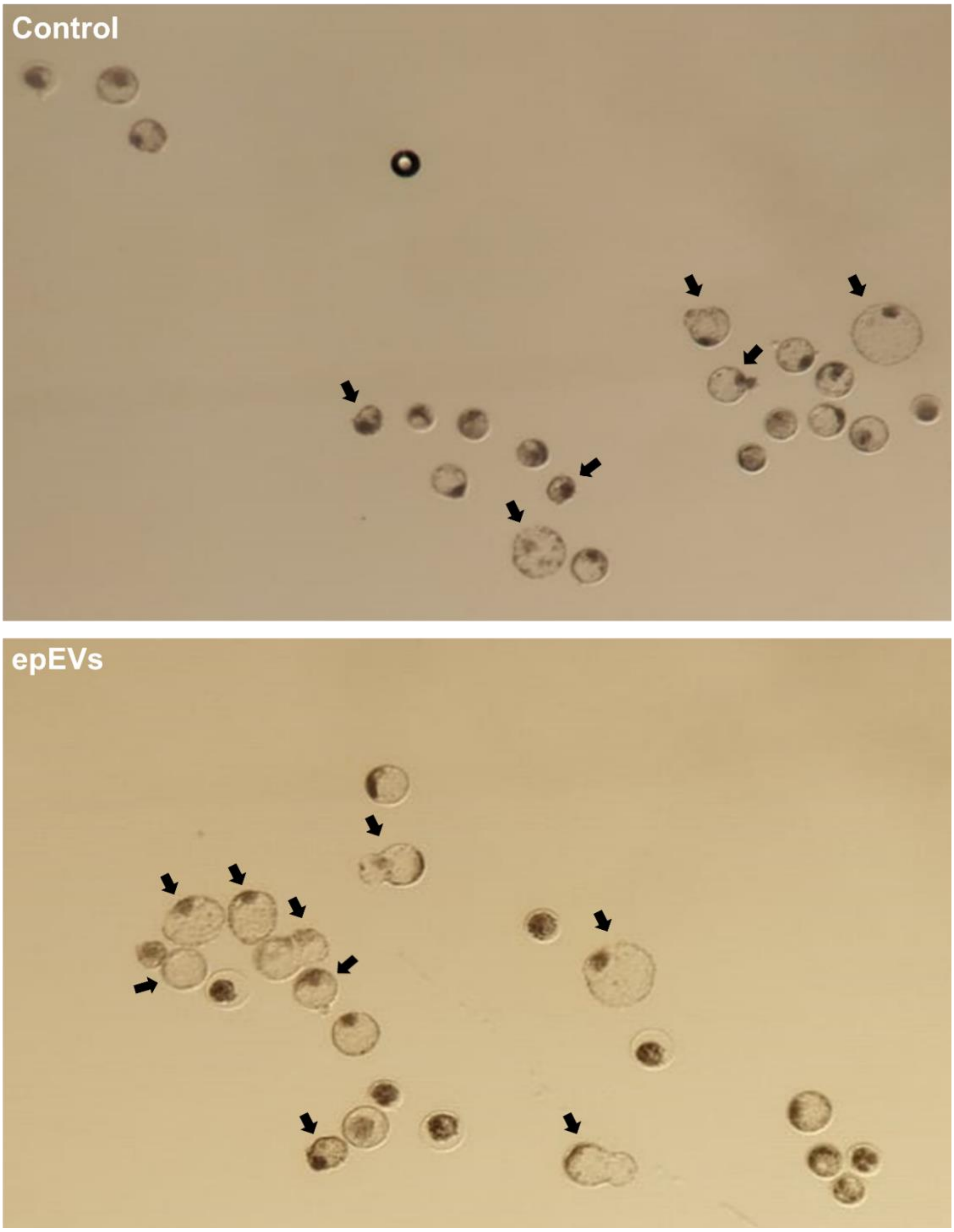

Supplement: Supplementary file 1 — Supporting File 1 [file MRD-93-e70138-s002.pdf]
