# Supplementary material for: Epididymosome‐Supplemented Extender Induces Changes in Morpho‐Functional Traits and microRNA Levels of Post‐Thaw Sperm and Improves Embryo Developmental Potential
Source: Mol Reprod Dev. 2026 Jul 30;93(8):e70138. doi: 10.1002/mrd.70138 (PMC13420828; doi:10.1002/mrd.70138)
Supplement: Supplementary file 4 — Supporting File 4 [file MRD-93-e70138-s001.docx]

**Title**

Epididymosome-supplemented extender induces changes in morpho-functional traits and microRNA levels of post-thaw sperm and improves embryo developmental potential

**Authors**

Laura Gabrielli Haupenthal^1^, Maria Alice Almeida^1^, Cibele Maria Prado^1^, Amanda Nespolo Silva^1^, Gabriela Melendes Schneider^1^, Paola Maria da Silva Rosa^1^, Flávio Vieira Meirelles^1^, Juliano Coelho da Silveira^1^, Felipe Perecin^1^, Maíra Bianchi Rodrigues Alves^2*^

**Author’s institutional affiliations**

^1^Department of Veterinary Medicine, School of Animal Science and Food Engineering, University of São Paulo, Pirassununga, São Paulo, Brazil.

^2^Department of Pathology, Theriogenology and One Healthy, School of Agricultural and Veterinary Sciences, São Paulo State University, Jaboticabal, São Paulo, Brazil.

**Supplementary Tables**

**Supplementary Table S1.** Mean, standard error of the mean (SEM) and P-value of the motility parameters evaluated on post-thaw sperm cryopreserved in the absence (Control Group) or presence (epEVs Group) of epididymosomes. Different letters indicate a statistical difference (P≤0.05). ^1^Hyperactive sperm according to Turri et al. (2011): VCL>70 μm/s, ALH>5 μm and VSL˂30%. ^2^Hyperactive sperm according to Ryu et al. (2019): VCL≥150 μm/s, ALH≥5 μm/s and VSL≤50%.

| Sperm motility parameters | Group | | *P-value* |
| --- | --- | --- | --- |
|  | Control (n=6) | EVs (n=6) |  |
| Total motility (%) | 30.73±3.49^b^ | 49.51±2.92^a^ | 0.0040 |
| Progressive motility (%) | 17.40±3.61^b^ | 31.73±3.70^a^ | 0.0007 |
| Rapid cells (%) | 24.56±3.12^b^ | 44.35±2.64^a^ | 0.0020 |
| Curvilinear velocity (VCL, μm/s), | 89.86±3.14^b^ | 105.35±5.09^a^ | 0.0275 |
| Straight line velocity (VSL, μm/s), | 45.00±3.72^b^ | 60.35±5.94^a^ | 0.0048 |
| Velocity average path (VAP, μm/s) | 64.78±1.91^b^ | 79.70±4.67^a^ | 0.0121 |
| Linearity (LIN, %), | 50.63±4.70^b^ | 57.43±5.23^a^ | 0.0322 |
| Straightness (STR, %), | 69.00±4.02^b^ | 74.95±3.67^a^ | 0.0090 |
| Wobble (WOB, %), | 72.53±3.12 | 75.88±3.58 | 0.1000 |
| Amplitude of lateral head displacement (ALH, μm/s), | 2.90±0.19^b^ | 3.25±0.18^a^ | 0.0189 |
| Beat cross frequency (BCF, Hz), | 7.96±0.50 | 8.81±0.28 | 0.0751 |
| Hiperactive sperm^1^ (%) | 3.05±0.90 | 5.36±2.84 | 0.3100 |
| Hiperactive sperm^2^ (%) | 3.70±0.76 | 8.16±3.29 | 0.1500 |

**Supplementary Table S2.** Sperm samples selected (in yellow) to miRNA analyzes based on similar motility in Control and epEVs group. The similar difference of motility between epEVs and Control group was also considered.

| **Animal** | **Group** | **Total motility*** | **Difference of motility (epEVs - Control)** |
| --- | --- | --- | --- |
| 1 | Control | 40.80 | 13.7 |
| 1 | epEVs | 54.50 |  |
|  |  |  |  |
| 2 | Control | 23.80 | 35 |
| 2 | epEVs | 58.80 |  |
|  |  |  |  |
| 3 | Control | 17.70 | 22 |
| 3 | epEVs | 39.70 |  |
|  |  |  |  |
| 4 | Control | 31.00 | 16.8 |
| 4 | epEVs | 47.80 |  |
|  |  |  |  |
| 5 | Control | 35.40 | 17.4 |
| 5 | epEVs | 52.80 |  |
|  |  |  |  |
| 6 | Control | 35.70 | 7.8 |
| 6 | epEVs | 43.50 |  |

*Evaluated by CASA.
